# Alternative regulatory mechanism for the maintenance of bone homeostasis via STAT5-mediated regulation of the differentiation of BMSCs into adipocytes

**DOI:** 10.1038/s12276-021-00616-9

**Published:** 2021-05-14

**Authors:** Semun Seong, Jung Ha Kim, Kabsun Kim, Inyoung Kim, Jeong-Tae Koh, Nacksung Kim

**Affiliations:** 1 Department of Pharmacology, Chonnam National University Medical School, Gwangju, 61469 Republic of Korea; 2 Hard-Tissue Biointerface Research Center, School of Dentistry, Chonnam National University, Gwangju, 61186 Republic of Korea; 3 Department of Pharmacology and Dental Therapeutics, School of Dentistry, Chonnam National University, Gwangju, 61186 Republic of Korea; 4 Department of Biomedical Sciences, Chonnam National University Medical School, Gwangju, 61469 Republic of Korea

**Keywords:** Growth factor signalling, Mechanisms of disease

## Abstract

STAT5 is a transcription factor that is activated by various cytokines, hormones, and growth factors. Activated STAT5 is then translocated to the nucleus and regulates the transcription of target genes, affecting several biological processes. Several studies have investigated the role of STAT5 in adipogenesis, but unfortunately, its role in adipogenesis remains controversial. In the present study, we generated adipocyte-specific *Stat5* conditional knockout (cKO) (*Stat5*^*fl/fl*^*;Apn-cre*) mice to investigate the role of STAT5 in the adipogenesis of bone marrow mesenchymal stem cells (BMSCs). BMSC adipogenesis was significantly inhibited upon overexpression of constitutively active STAT5A, while it was enhanced in the absence of *Stat5* in vitro. In vivo adipose staining and histological analyses revealed increased adipose volume in the bone marrow of *Stat5* cKO mice. ATF3 is the target of STAT5 during STAT5-mediated inhibition of adipogenesis, and its transcription is regulated by the binding of STAT5 to the *Atf3* promoter. ATF3 overexpression was sufficient to suppress the enhanced adipogenesis of *Stat5-*deficient adipocytes, and *Atf3* silencing abolished the STAT5-mediated inhibition of adipogenesis. *Stat5* cKO mice exhibited reduced bone volume due to an increase in the osteoclast number, and coculture of bone marrow-derived macrophages with *Stat5* cKO adipocytes resulted in enhanced osteoclastogenesis, suggesting that an increase in the adipocyte number may contribute to bone loss. In summary, this study shows that STAT5 is a negative regulator of BMSC adipogenesis and contributes to bone homeostasis via direct and indirect regulation of osteoclast differentiation; therefore, it may be a leading target for the treatment of both obesity and bone loss-related diseases.

## Obesity and osteoporosis: a common therapeutic target

A protein connected with bone maintenance and fat cell differentiation could provide a novel therapeutic target for both obesity and osteoporosis. The processes of healthy bone remodeling and fat cell (adipocyte) differentiation from bone marrow stem cells (BMSCs) are intrinsically connected. The transcription factor protein STAT5 plays roles in maintaining bone homeostasis and adipocyte differentiation, but its role in the latter is unclear. Nacksung Kim at Chonnam National University Medical School in Gwangju, South Korea, and co-workers examined the role of STAT5 in mice. Mice without the *Stat5* gene had increased fat tissue in their bone marrow, suggesting increased BMSC differentiation into adipocytes. The mice also had reduced bone mass due to increased numbers of bone-degrading cells. Further investigations showed that STAT5 regulates the differentiation of BMSCs into adipocytes via activation of a regulatory gene.

## Introduction

There are seven known members of the signal transducer and activator of transcription factor (STAT) protein family: STAT1, STAT2, STAT3, STAT4, STAT5A, STAT5B, and STAT6. STAT proteins are involved in numerous biological processes, such as cell proliferation, differentiation, apoptosis, milk production, and immunity^1–4^. Activation of STAT protein family members is usually associated with Janus kinase (JAK). Upon stimulation by various cytokines, growth factors, and hormones, JAK is activated, and activated JAK further phosphorylates STAT proteins on tyrosine residues^5,6^. Subsequently, phosphorylated or activated STAT proteins form dimers and translocate to the nucleus, where they bind to specific sites called GAS elements (TTCNNNGAA) to regulate the transcription of target genes^7,8^.

Among the many STAT protein family members is STAT5, which has two isoforms, STAT5A and STAT5B. Although these isoforms are encoded by separate genes, they have over 90% amino acid sequence similarity and may perform redundant functions^9^. Previously, we demonstrated that STAT5 contributes to the maintenance of bone homeostasis by mediating the inhibitory effect of IL-3 on osteoclastogenesis. When *Stat5* was knocked down specifically in bone marrow macrophages using Mx1-cre, evaluation of the in vivo bone phenotype revealed that *Stat5*^*fl/fl*^*;Mx1-cre* mice exhibit an osteoporotic bone phenotype due to an increase in the osteoclast number rather than a change in the osteoblast number^10^. Interestingly, we observed that there was a significant increase in the number of adipocytes located in the bone marrow of *Stat5*^*fl/fl*^*;Mx1-cre* mice in a previous study (Supplementary Fig. 1).

Indeed, the function of STAT5 is not restricted to bone; it also contributes to adipocyte differentiation. Although several studies have investigated the function of STAT5 in adipocyte differentiation, the observed role of STAT5 remains controversial. Previously, a positive role for STAT5 in adipocyte differentiation was reported; it was observed that silencing and overexpression of STAT5 resulted in inhibition and promotion adipocyte differentiation, respectively^11–14^. In contrast, a negative role for STAT5 in adipocyte differentiation has been identified in several studies through establishment of models of adipocyte-specific *Stat5* conditional deficiency and growth hormone treatment experiments. In these experiments, it was observed that *Stat5* deficiency resulted in an increase in subcutaneous adipose tissue mass due to an increase in the adipocyte number in *Stat5* conditional knockout (cKO) mice and to lipid accumulation in the liver and muscles in *Stat5*^*fl/fl*^*;Alb-cre* and *Stat5*^*fl/fl*^*;Myf5-cre* mice, respectively^15–17^. In addition, it was observed that growth hormone inhibits adipocyte differentiation by activating STAT5^18^. The studies showing a positive role for STAT5 in adipocyte differentiation were primarily performed using 3T3-L1 cells, while the studies showing a negative role were performed with non-cell line cells such as bovine preadipocytes or with adipose or liver tissues from mice with adipocyte-specific or liver-specific *Stat5* cKO, respectively.

Notably, both osteoclasts and adipocytes influence bone homeostasis. Osteoclasts, well-known bone-resorbing cells, contribute to the maintenance of bone mass by participating in bone remodeling^19^. Disruptions in osteoclast function, such as excessive or defective resorption, result in imbalances between bone formation and bone resorption and can induce bone-related diseases such as osteoporosis and osteopetrosis^20,21^. Moreover, adipocytes exhibit correlations with osteoblasts and osteoclasts. It has been reported that the number of bone marrow adipocytes increases with aging and that there is a correlation between an increasing adipocyte number and the occurrence of bone diseases such as osteoporosis^22,23^. Previously, Mori et al.^24^ reported that when osteoclast precursor cells were cocultured with either adipocytes or mesenchymal stem cells, enhanced osteoclast differentiation was observed in an adipocyte coculture system. In addition, adipocytes derived from bone marrow mesenchymal stem cells (BMSCs) could be a major factor involved in osteoblast differentiation. Due to the similar origins of adipocytes and osteoblasts, their differentiation is reciprocally regulated; when the lineage favors adipogenic differentiation, the result is a decreased osteoblast number, and such imbalances are more often observed with age-induced bone loss^25,26^. In addition, Liu et al.^27^ reported that coculture of osteoblasts with adipocytes resulted in decreased expression of Runx2 in osteoblasts at both the mRNA and protein levels. Since Runx2 is an indispensable factor for osteoblast differentiation, this previous study suggests that pre-existing adipocytes may negatively regulate the differentiation of newly formed osteoblasts.

*Stat5*^*fl/fl*^*;Mx1-cre* mice in the previous study simultaneously exhibited decreased bone mass and an increased number of bone marrow adipocytes, strongly suggesting that STAT5 may act as a negative regulator of adipocyte differentiation in bone marrow. Therefore, we performed an investigation using adipocyte-specific *Stat5* cKO mice (*Stat5*^*fl/fl*^*;Apn-cre*) to clarify the role of STAT5 in adipogenesis of BMSCs and the effect of STAT5-regulated adipocyte differentiation of BMSCs in bone. The present study shows that STAT5 inhibits adipogenic differentiation by activating *Atf3*, a known negative regulator of adipocyte differentiation. Furthermore, the results of both in vitro and in vivo experiments revealed a negative role for STAT5 in adipocyte differentiation of BMSCs in bone marrow; therefore, we suggest that STAT5 plays an important role in maintaining bone homeostasis by regulating the differentiation of BMSCs into adipocytes.

## Materials and methods

### Reagents

Recombinant human RANKL was purified from bacteria. Recombinant human M-CSF was a gift from Dr. Daved Fremont (Washington University, St. Louis, MO, USA). Insulin, rosiglitazone, dexamethasone, and 3-isobutyl-1-methylxanthine (IBMX) were purchased from Sigma-Aldrich (St. Louis, MO, USA).

### Cell culture, differentiation, and staining

BMSCs were isolated as previously described with modifications^28^. Briefly, femurs and tibias were flushed with ɑ-MEM after harvesting from mice and were then cultured in a 10 cm culture dish containing ɑ-MEM (HyClone Laboratories, Logan, UT, USA) supplemented with 10% FBS (HyClone Laboratories), 100 U/mL penicillin, and 100 mg/mL streptomycin (Life Technologies, Carlsbad, CA, USA). After 3 days, nonadherent cells were removed by replacing the culture medium and were further cultured to confluence. When the cells were confluent, they were split at a 1:2 ratio and further cultured to confluence; passage 2 BMSCs were used for experiments.

When the cells were confluent, they were detached using 0.05% trypsin, plated in 96-well plates or 6-well plates at a density of 3 × 10^4^ cells/well or 7 × 10^5^ cells/well, respectively, and further cultured with insulin (10 µg/ml), rosiglitazone (1 µM), dexamethasone (0.1 µM), and IBMX (0.5 mM). After 3 days, the medium was replaced with medium containing insulin alone, and the cells were cultured for 3 more days. Cultured cells were fixed and stained using Oil Red O solution (Sigma-Aldrich) or Nile Red solution (Thermo Fisher Scientific, Waltham, MA, USA). Oil Red O-positive adipocytes were counted.

Adipose tissue-derived mesenchymal stem cells (ADSCs) were obtained by mechanical and enzymatic digestion of biopsied adipose tissue. Inguinal adipose tissues were mechanically minced using scissors prior to enzymatic digestion in HBSS (Life Technologies) containing 0.05% collagenase type I (Life Technologies). Digested adipose tissues were neutralized at a 1:2 volumetric ratio in ɑ-MEM containing 10% FBS and filtered through a filter with a pore size of 100 µm. Filtered cells were fractionated by centrifugation to isolate the stromal vascular fraction. The isolated stromal vascular fraction was filtered through a filter with a pore size of 40 µm and cultured to confluence in ɑ-MEM (HyClone Laboratories) containing 20% FBS (HyClone Laboratories) and 1% GlutaMAX (Life Technologies) in a 10 cm culture dish; passage 2 ADSCs were used for experiments.

Osteoclasts were obtained from murine bone marrow cells according to a previous study. In brief, bone marrow cells were obtained by flushing femurs and tibias with ɑ-MEM (HyClone Laboratories). The isolated cells were incubated with α-MEM (HyClone Laboratories) containing 10% FBS (HyClone Laboratories), 100 U/mL penicillin, 100 mg/mL streptomycin (Life Technologies), and 30 ng/mL M-CSF for 3 days. Nonadherent cells were removed, and the remaining adherent cells were used for osteoclast differentiation by plating in 96-well plates at a density of 1 × 10^4^ cells per well in culture medium containing M-CSF (30 ng/mL) and RANKL (100 ng/mL). Mature osteoclasts were fixed and stained for TRAP. TRAP-positive multinuclear cells with more than three nuclei were considered positive and counted as osteoclasts.

### Mice

*Stat5* WT (*Stat5*^*fl/fl*^) mice with both *Stat5a* and *Stat5b* targeted on a C57BL/6 background were generated as previously described^1^. *Apn-cre*^*+/*−^ mice on a C57BL/6 background expressing Cre recombinase under the control of the *Adipoq* promoter were generated as previously described^29^. *Stat5* cKO (*Stat5*^*fl/fl*^*;Apn-cre*) mice were generated by crossing *Stat5* floxed (*Stat5*^*fl/fl*^) mice with *Apn-cre*^*+/*−^ mice. *Stat5*^*fl/fl*^ mice were crossed with *Apn-cre*^*+/*−^ mice to obtain *Stat5*^*fl/+*^*;Apn-cre*^*+/*−^ offspring. The offspring were backcrossed with *Stat5*^*fl/fl*^ mice to obtain *Stat5*^*fl/fl*^*;Apn-cre*^*+/−*^ mice. Genotyping was performed with genomic DNA isolated from tails by PCR using the following primers: *Stat5* WT forward, 5′-GAA AGC ATG AAA GGG TTG GAG-3′; *Stat5* WT reverse, 5′-AGC AGC AAC CAG AGG ACT TAC-3′; *Stat5* fl2 forward, 5′-TAC CCG CTT CCA TTG CTC AG-3′; *Stat5* fl2 reverse, 5′-AGC AGC AAC CAG AGG ACT AC-3′; *Apn-cre* forward, 5′-GCC TGC ATT ACC GGT CGA TGC AAC GA-3′; and *Apn-cre* reverse, 5′-GTG GCA GAT GGC GCG GCA ACA CCA TT-3′. For experiments, male littermates that were maintained in the same cage and consumed the same quantity and quality of food and water as the genetically modified mice were used to ensure objectiveness in comparisons and observations of the effects of the genotypes. All animal experiments were approved by the Chonnam National University Medical School Research Institutional Animal Care and Use Committee and were carried out in accordance with approved guidelines.

### Plasmid DNA constructs

The full-length coding sequence of mouse *Atf3* (NM_007498.3) was cloned from mature osteoclasts by digestion with *EcoRI* and *XhoI* and subcloned into a retroviral vector (*pMX-IRES-EGFP*) containing a Flag tag at the C-terminal end of the cloning site. The constitutively active *Stat5a* (pMX-STAT5A1*6-IRES-EGFP) expression vector was constructed as previously described^30^.

### Retroviral transduction

Plat E cells were transfected in the presence of FuGENE 6 (Promega, Madison, WI, USA) to produce packaged retroviruses according to the manufacturer’s instructions. The retroviral supernatant was collected 48 h after transfection and used as the medium for incubating the cells of interest for 6 h in the presence of 10 μg/mL polybrene (Sigma-Aldrich).

### Quantitative real-time PCR

qPCR was performed in triplicate using SYBR Green (Qiagen, GmbH, Hilden, Germany) and Rotor-Gene Q (Qiagen) with specific primers. The mRNA expression levels of the analyzed genes were normalized to the expression level of *Gapdh*. The relative quantified value for the expression of each target gene compared to that of the calibrator for that target gene was expressed as 2^−(Ct − Cc)^ (where Ct and Cc are the mean threshold cycle differences of the target gene and the calibrator gene, respectively, after normalization to the expression level of *Gapdh*). The relative expression levels for each sample are presented on a semilog plot. The primer sequences were as follows:

*Gapdh* forward, 5′-TGA CCA CAG TCC ATG CCA TCA CTG-3′; *Gapdh* reverse, 5′-CAG GAG ACA ACC TGG TCC TCA GTG-3′; *c-fos* forward, 5′-ATG GGC TCT CCT GTC AAC ACA CAG-3′; *c-fos* reverse, 5′-TGG CAA TCT CAG TCT GCA ACG CAG-3′; *Nfatc1* forward, 5′-CTC GAA AGA CAG CAC TGG AGC AT-3′; *Nfatc1* reverse, 5′-CGG CTG CCT TCC GTC TCA TAG-3′; *Acp5* forward, 5′-CTG GAG TGC ACG ATG CCA GCG ACA-3′; *Acp5* reverse, 5′-TCC GTG CTC GGC GAT GGA CCA GA-3′; *Pparɣ* forward, 5′-TCC AGC ATT TCT GCT CCA CA-3′; *Pparɣ* reverse, 5′-ACA GAC TCG GCA CTC AAT GG-3′; *Cebpα* forward, 5′-AAG AAG TCG GTG GAC AAG AAC AG-3′; *Cebpα* reverse, 5′-TGC GCA CCG CGA TGT-3′; *Fabp4* forward, 5′-AAA TCA CCG CAG ACG ACA −3′; *Fabp4* reverse, 5′-CAC ATT CCA CCA CCA GCT-3′; *Stat5a* forward, 5′-AGT ATT ACA CTC CTG TAC TTG CGA AAG-3′; *Stat5a* reverse, 5′-GGA GCT TCT AGC GGA GGT GAA GAG ACC-3′; *Stat5b* forward, 5′-GGT CCC CTG TGA GCC CGC AAC-3′; *Stat5b* reverse, 5′-TGA CTG TGC GTG AGG GAT CCA CTG ACT-3′; *Atf3* forward, 5′-AAC AAC AGA CCC CTG GAG ATG TC-3′; *Atf3* reverse, 5′-TCC TCA ATC TGG GCC TTC AGC TC-3′; *Rankl* forward, 5′-CCT GAG ACT CCA TGA AAA CGC-3′; *Rankl* reverse, 5′-TCG CTG GGC CAC ATC CAA CCA TGA-3′; *Opg* forward, 5′-CAG TGA TGA GTG TGT GTA TTG CAG-3′; and *Opg* reverse, 5′-TTA TAC AGG GTG CTT TCG ATG AAG-3′.

### Chromatin immunoprecipitation (ChIP)

ChIP was performed using an EZ-ChIP™ kit (Millipore, Burlington, MA, USA) according to the manufacturer’s instructions. In brief, BMSCs were isolated from *Stat5* cKO mice and their wild-type littermates. Protein and DNA in the cells were crosslinked, DNA was then sheared by sonication. Protein/DNA complexes were immunoprecipitated with IgG or an anti-pSTAT5 antibody (Cell Signaling Technology, Danvers, MA, USA). Subsequently, protein/DNA complexes were eluted and subjected to reverse crosslinking, and DNA was purified using spin columns provided with the EZ-ChIP™ kit (Millipore). Purified DNA was amplified by PCR. The sequences of the primers containing the STAT5-binding sites in the *Atf3* promoter region were as follows. −16-*Atf3* forward, 5′-ACC GCC CCT TCT CGC ACT TG-3′; −16-*Atf3* reverse, 5′-GCG CGT TGC ATC ACC CCT TT-3′; −239-*Atf3* forward, 5′-CAC CCC CCC TTC CCC AAC CT-3′; −239-*Atf3* reverse, 5′-CTG CGT TCC TCG CAC GCC CG-3′; −852-*Atf3* forward, 5′-TAG AGC CCT CTG TTC CTG CA-3′; −852-*Atf3* reverse, 5′-AGG AAA GAT TTG GAG AGA TC-3′; −1332 (negative control binding site)-*Atf3* forward, 5′-CCT GTG AAG GGC CAG ACT CT-3′; and −1332 (negative control binding site)-*Atf3* reverse, 5′-GAA GGC GAT CAG AAG GTC AC-3′. The final products were analyzed by 4% agarose gel electrophoresis.

### Western blotting

Cells were washed with phosphate-buffered saline (PBS) and lysed with lysis buffer [50 mM Tris-HCl (pH 8.0), 150 mM NaCl, 1 mM EDTA, 0.5% Nonidet P-40, 1 mM PMSF, and protease inhibitor cocktails]. Proteins in the cell lysates were separated by SDS-PAGE and transferred onto a PVDF membrane (Millipore). The membrane was blocked with 5% skim milk in TBS-T [10 mM Tri-HCl (pH 7.6), 150 mM NaCl, and 0.1% Tween 20] and immunoblotted with antibodies against STAT5A (Santa Cruz Biotechnology, Dallas, TX, USA), STAT5B (Cell Signaling Technology), and Actin-HRP (Sigma-Aldrich). Signals were detected with ECL solution (Millipore) and analyzed using an Azure c300 luminescent image analyzer (Azure Biosystems, Dublin, CA, USA).

### Luciferase reporter assay

The −1850-*Atf3-*Luc construct was a gift from J-T.K. (Department of Pharmacology and Dental Therapeutics, School of Dentistry, Chonnam National University, South Korea). First, 293T cells were transfected with −1850-*Atf3*-Luc using FuGENE 6 (Promega) according to the manufacturer’s instructions. Subsequently, luciferase activity was measured in duplicate with a dual-luciferase reporter assay system (Promega).

### siRNA transfection

BMSCs were transfected with Lipofectamine 2000 (Life Technologies) to deliver *Atf3* siRNA (*siAtf3*) according to the manufacturer’s instructions. Transfected BMSCs were incubated at 37 °C for 4 h, and the medium was then replaced with growth medium or adipogenic differentiation medium.

### Osmium tetroxide staining

Femurs from 24-week-old *Stat5* cKO mice and their wild-type littermates were isolated and fixed with 4% paraformaldehyde overnight, followed by decalcification in 3.8% formaldehyde buffer containing 5.5% EDTA for 3 weeks at 4 °C. The proximal portions of femurs were cut and incubated with 1 mL of osmium tetroxide solution containing 1% osmium tetroxide and 2.5% potassium dichromate for 48 h. After 48 h, osmium tetroxide-stained femurs were washed with distilled water for 2 h and subjected to µCT analysis. The obtained raw μCT data were analyzed in a blinded manner, and the investigator had no knowledge of the genotypes of the subjects until the final processed data were analyzed. Three-dimensional images of trabecular bones were remodeled with a kernel density ranging from 220 to 250 using Ant software (Skyscan). Different batches of littermate subjects were analyzed, and a representative set was used.

### Isolation of bone marrow adipose cells

Bone marrow adipose cells were isolated as previously described^31^ with some modifications. Briefly, the edges of long bones were cut, and bone marrow was extracted by rapid centrifugation (9000 × *g* for 10 s) and digested with 0.2% collagenase (Thermo Fisher Scientific) for 30 min at 37 °C. Subsequently, the cells were filtered through a 100-µm cell strainer and subjected to centrifugation at 1700 × *g* for 5 min. The floating adipocytes were collected and washed with PBS before use.

### Micro-computed tomography (µCT)

Femurs from 8-week-old *Stat5* cKO mice and their wild-type littermates were harvested and subjected to μCT, and images were acquired using a Skyscan 1172 system (Skyscan, Kontich, Belgium) with the following conditions: X-ray source at 50 kV and 201 μA with a 0.5 mm aluminum filter. Images were acquired at increments of 0.7 degrees, and the raw images for all samples were reconstructed into serial cross-sectional images with identical thresholds (0–6000 in Hounsfield units) using Recon software (Skyscan). CTAn software (Skyscan) was used to manually draw a region of interest on the reconstructed images within 300 steps of the trabecular bone of the distal femur, beginning 80 steps away from the epiphyseal plate. Three-dimensional morphometric analysis was performed by measuring the bone volume fraction (BV/TV), trabecular thickness (Tb.Th), trabecular separation (Tb.Sp), and trabecular number (Tb.N). The obtained raw μCT data were processed in a blinded manner, and the investigator had no knowledge of the genotypes of the subjects until the final processed data were analyzed. Three-dimensional images of trabecular bones were remodeled with a kernel density ranging from 90 to 165 using Ant software (Skyscan). Different batches of littermate subjects were analyzed, and a representative set was used.

### Histological analysis

Tibias were harvested from 8-week-old *Stat5* cKO mice and their wild-type littermates and fixed with 4% paraformaldehyde overnight, followed by decalcification in 3.8% formaldehyde buffer containing 5.5% EDTA for 3 weeks at 4 °C. Decalcified tibias were dehydrated and embedded in paraffin blocks. The paraffin blocks were cut into 4-μm-thick longitudinal sections. The sectioned samples were deparaffinized using xylene and subjected to tartrate-resistant acid phosphatase (TRAP) staining to identify osteoclasts located below the growth plate and hematoxylin & eosin (H&E) staining to identify osteoblasts located below the growth plate and adipocytes located below the bone marrow. To ensure a blind analysis, the investigator quantified osteoclasts, osteoblasts, and adipocytes in the stained, sectioned samples in a blinded manner, with no knowledge of the genotypes of the subjects until the final processed data were analyzed.

### Statistical analysis

All values are expressed as the means ± standard deviations (SDs). Statistical significance was determined by using two-tailed Student’s *t* tests for two independent samples or analysis of variance with post hoc Tukey’s HSD test for multiple group comparisons. *P* < 0.05 was considered statistically significant.

## Results

### STAT5 plays a negative role in adipogenic differentiation of BMSCs

Previously, we reported that mice with bone marrow-derived macrophage-specific *Stat5* cKO (*Stat5*^*fl/fl*^*;Mx1-cre*) exhibit an osteoporotic bone phenotype due to an increased osteoclast number^10^. Unexpectedly, we also observed that there was a significant increase in the adipocyte number in the bone marrow of *Stat5*^*fl/fl*^*;Mx1-cre* mice (Supplementary Fig. 1a, b). Since Mx1-cre is generally used to knockout genes of interest specifically in macrophages and there was an alteration in the number of adipocytes derived from BMSCs in *Stat5*^*fl/fl*^*;Mx1-cre* mice, a question was raised as to whether Mx1-cre is able to knockdown both *Stat5a* and *Stat5b* in BMSCs. To answer this question, the mRNA expression of *Stat5a* and *Stat5b* throughout the adipogenic differentiation of BMSCs from *Stat5*^*fl/fl*^*;Mx1-cre* mice was analyzed. We observed that the mRNA expression of both *Stat5a* and *Stat5b* was significantly suppressed beginning at the mesenchymal stem cell stage (Supplementary Fig. 1c). Therefore, we postulated that STAT5 may be involved in adipogenic differentiation of BMSCs.

**Fig. 1:**
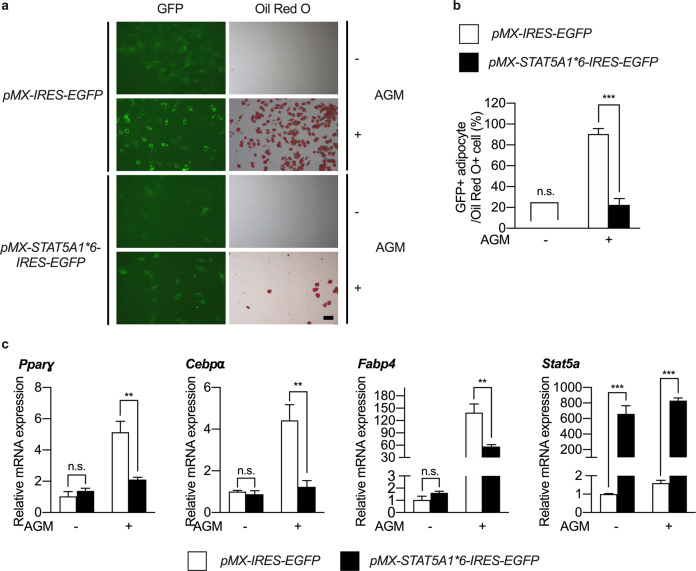
Ectopic expression of STAT5A inhibits BMSC adipogenesis in vitro. **a–c** BMSCs were transduced with control (*pMX-IRES-EGFP*) or constitutively active STAT5A (pMX-STAT5A1*6-IRES-EGFP) retroviral vectors and cultured in the presence or absence of adipogenic differentiation factors (insulin, rosiglitazone, dexamethasone, and IBMX) for 5 days. **a** Cultured cells were stained using Oil Red O solution and Nile Red solution. **b** The ratio of GFP/Oil Red O double-positive adipocytes to Oil Red O-positive adipocytes was calculated. ***P < 0.001 vs. control; n.s. not significant. Statistical analyses were performed via Student’s *t* test. **c** The mRNA levels of *Pparɣ*, *Cebpα*, *Fabp4*, and *Stat5a* were assessed by quantitative real-time PCR. The data are presented as the mean ± SD of triplicate samples. **P < 0.01; ***P < 0.001 vs. control; n.s. not significant. Statistical analyses were performed via Student’s *t* test. AGM Adipogenic differentiation medium. Bar: (**a**) 100 µm.

To investigate the role of STAT5 in adipogenic differentiation of BMSCs, BMSCs were isolated from the long bones of 5-week-old ICR mice, transduced with either the control or constitutively active STAT5 plasmid, and differentiated into adipocytes. We observed that there was a reduction in the number of Oil Red O-positive adipocytes upon STAT5 overexpression (Fig. 1a). To more accurately examine the inhibitory effect of STAT5, we evaluated the ratio of GFP/Oil Red O double-positive adipocytes to Oil Red O-positive adipocytes. As shown in Fig. 1b, STAT5 overexpression significantly reduced this ratio, which indicated that the cells were successfully transduced with STAT5 and exhibited suppression of adipogenic differentiation. Furthermore, when the mRNA expression of adipogenic marker genes such as *Pparɣ*, *Cebpɑ*, and *Fabp4* was analyzed by real-time PCR, we observed that the mRNA expression of these marker genes was strongly reduced upon STAT5 overexpression (Fig. 1c).

**Fig. 2:**
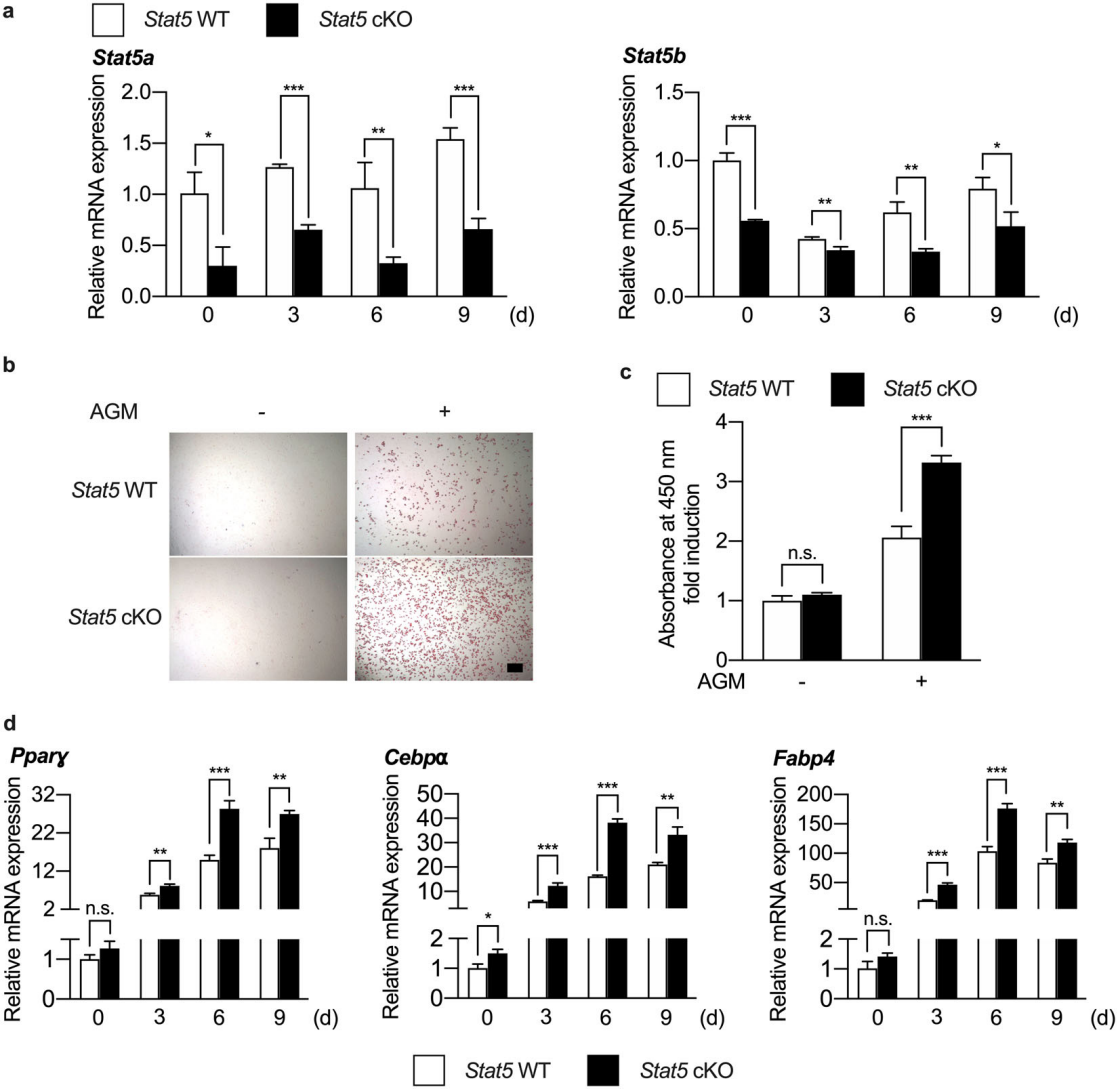
BMSC adipogenesis is enhanced in the absence of *Stat5* in vitro. **a–d** BMSCs were isolated from femurs and tibias of *Stat5* cKO mice and their wild-type littermates and were cultured in the presence or absence of adipogenic differentiation factors (insulin, rosiglitazone, dexamethasone, and IBMX) for the indicated times. **a** The mRNA levels of *Stat5a* and *Stat5b* were assessed by quantitative real-time PCR. *P < 0.05; **P < 0.01; ***P < 0.001 vs. control. Statistical analyses were performed using Student’s *t* test. **b** Cultured cells were stained using Oil Red O solution. **c** Quantification of Oil Red O-stained cells isolated using isopropanol. ***P < 0.001 vs. control; n.s. not significant. Statistical analyses were performed using Student’s *t* test. **d** The mRNA levels of *Pparɣ*, *Cebpα*, and *Fabp4* were assessed by quantitative real-time PCR. The data are presented as the mean ± SD of triplicate samples. *P < 0.05; **P < 0.01; ***P < 0.001 vs. control; n.s. not significant. Statistical analyses were performed via Student’s *t* test. AGM Adipogenic differentiation medium. Bar: (**b**) 200 µm.

Since ectopic expression of STAT5 suppressed the adipogenic differentiation of BMSCs, it was necessary to examine the opposite condition, i.e., STAT5 deficiency. Adipocyte-specific *Stat5* cKO (*Stat5*^*fl/fl*^*;Apn-cre*) mice were generated, and BMSCs were isolated from the long bones of *Stat5* cKO mice and their wild-type littermates and were differentiated into adipocytes. First, knockdown of *Stat5a* and *Stat5b* was assessed by real-time PCR and western blotting. The mRNA expression levels of both *Stat5a* and *Stat5b* and the protein expression levels of STAT5A and STAT5B were significantly reduced in *Stat5* cKO cells (Fig. 2a and Supplementary Fig. 2). Having confirmed that *Stat5a* and *Stat5b* were knocked down in *Stat5* cKO adipocytes, we analyzed the effect of *Stat5* deficiency on adipogenic differentiation of BMSCs. There was an increase in the number of Oil Red O-positive adipocytes in the absence of *Stat5* (Fig. 2b, c). Furthermore, the mRNA expression of adipogenic marker genes was significantly upregulated in *Stat5* cKO adipocytes (Fig. 2d). Therefore, it could be deduced that STAT5 acts as a negative regulator of adipogenic differentiation of BMSCs in vitro.

### Bone marrow adiposity is increased in adipocyte-specific *Stat5* cKO mice

**Fig. 3:**
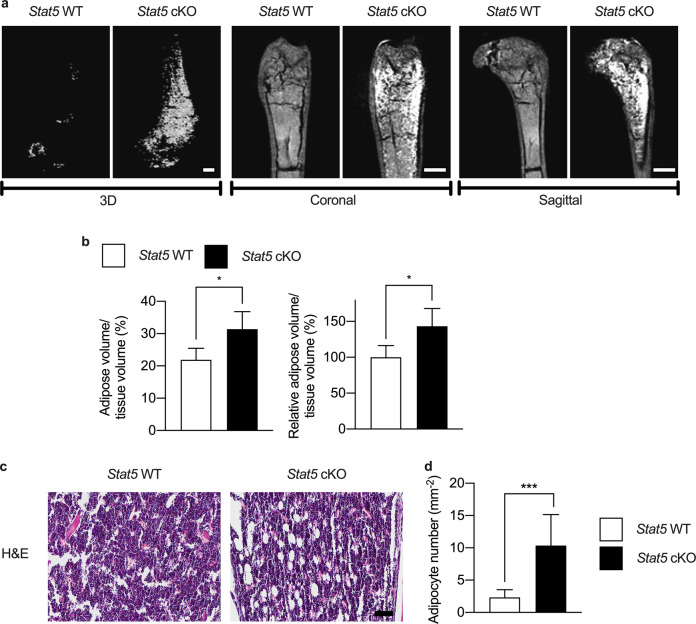
Bone marrow adiposity is increased in *Stat5* cKO mice. **a**, **b** Femurs isolated from *Stat5* cKO mice (*n* = 4) and their wild-type littermates (*n* = 3) were subjected to osmium/tetroxide staining. **a** Representative images of osmium/tetroxide-stained femurs isolated from *Stat5* cKO mice and their wild-type littermates. **b** Quantification of osmium tetroxide-stained bone marrow adipose tissue based on micro-CT analysis. *P < 0.05 vs. control. Statistical analyses were performed via Student’s *t* test. **c**, **d** Tibias harvested from *Stat5* cKO mice and their wild-type littermates were subjected to hematoxylin & eosin staining (*n* = 10 per group). **c** Representative images of hematoxylin & eosin-stained tibias harvested from *Stat5* cKO mice and their wild-type littermates. **d** Quantification of bone marrow adipose tissue based on hematoxylin & eosin staining. ***P < 0.001 vs. control. Statistical analyses were performed via Student’s *t* test. Bars: (**a**) 500 µm; (**c**) 100 µm.

The effect of *Stat5* deficiency on adipogenic differentiation of BMSCs was further investigated in vivo. Femurs and tibias were harvested from *Stat5* cKO mice and their wild-type littermates and examined via osmium tetroxide staining and histological analysis. 3D, coronal, and sagittal images of osmium tetroxide-stained femurs revealed that there was an increase of ~43% in the bone marrow adipose volume in the femurs of *Stat5* cKO mice (Fig. 3a, b). This increase in bone marrow adipose tissue in *Stat5* cKO mice was further confirmed by histological analysis. H&E staining revealed a significant increase in the bone marrow adipocyte numbers in *Stat5* cKO mice (Fig. 3c, d). Furthermore, increases in the size and mass of adipose tissue were observed in inguinal adipose tissues from *Stat5* cKO mice, indicating that adipocyte hypertrophy occurred due to *Stat5* deficiency and resulted in an increased adipose tissue mass (Supplementary Fig. 3a, b). In addition to in vivo adipose tissue analysis, in vitro experiments using ADSCs revealed an increase in Oil Red O-positive adipocytes and increases in the mRNA expression levels of *Pparɣ*, *Cebpa*, and *Fabp4* in the absence of *Stat5* (Supplementary Fig. 3c–e). The positive effect of *Stat5* deficiency on adipogenic differentiation of BMSCs that we observed was consistent with the results of the in vitro experiment shown in Fig. 2. These results show that adipocyte-specific *Stat5* cKO mice exhibit increased bone marrow adiposity, which further supports the idea that STAT5 plays a negative role in adipogenic differentiation of BMSCs.

### ATF3 mediates the inhibitory effect of STAT5 on adipogenic differentiation of BMSCs

**Fig. 4:**
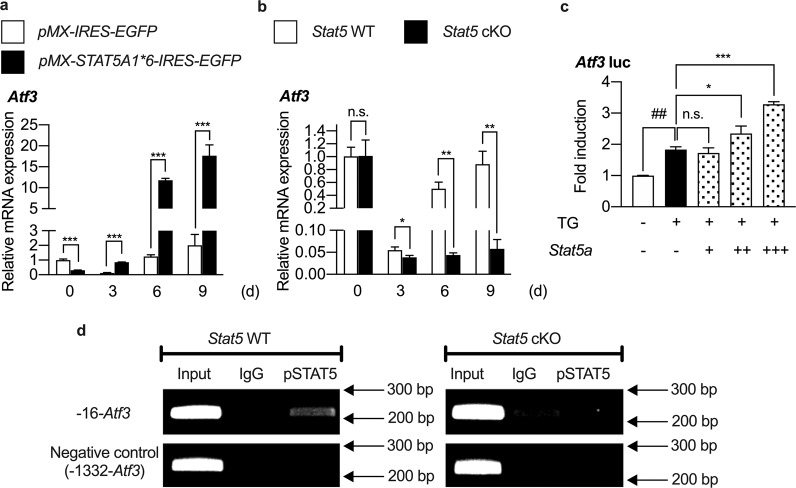
*Atf3* expression is regulated by STAT5 at the transcriptional level. **a** BMSCs were transduced with control (*pMX-IRES-EGFP*) or constitutively active STAT5A (pMX-STAT5A1*6-IRES-EGFP) retroviral vectors and cultured in the presence or absence of adipogenic differentiation factors (insulin, rosiglitazone, dexamethasone, and IBMX) for the indicated days, followed by assessment of mRNA levels by quantitative real-time PCR. The data are presented as the mean ± SD of triplicate samples. ***P < 0.001 vs. control. Statistical analyses were performed using Student’s *t* test. **b** BMSCs were isolated from the femurs and tibias of *Stat5* cKO mice and their wild-type littermates and were cultured in the presence or absence of adipogenic differentiation factors (insulin, rosiglitazone, dexamethasone, and IBMX) for the indicated days, followed by assessment of mRNA levels by quantitative real-time PCR. The data are presented as the mean ± SD of triplicate samples. *P < 0.05; **P < 0.01 vs. control; n.s., not significant. Statistical analyses were performed via Student’s *t* test. **c** The *Atf3* luciferase reporter plasmid (20 ng) was transfected into 293T cells, and the cells were treated with thapsigargin (TG) (10 nM) and increasing amounts of *Stat5a* (100, 300, and 500 ng). Luciferase activity was measured using a dual-luciferase reporter assay system. The data are presented as the mean ± SD of duplicate samples. ^##^*P* < 0.01 vs. the negative control not treated with either TG or *Stat5a*; *P < 0.05; ***P < 0.001 vs. the positive control treated with TG but not with *Stat5a*; n.s. not significant. Statistical analyses were performed via two-way ANOVA. **d** Chromatin immunoprecipitation of pSTAT5 and the *Atf3* promoter. Immunoprecipitation was performed using anti-pSTAT5 antibodies or IgG as the negative control. Precipitated DNA was subjected to PCR with primers targeting the STAT5-binding site (−16-*Atf3*) and a negative control binding site (−1332-*Atf3*).

To investigate the mechanism underlying the inhibitory effect of STAT5 on adipogenic differentiation of BMSCs, it was necessary to identify the target of STAT5. In our previous study^10^, there were several genes whose expression depended on the presence of *Stat5*. Among the various genes, *Atf3* was the gene whose expression was most significantly regulated by STAT5 (GSE76988). Since ATF3 is known to inhibit adipogenic differentiation^32,33^, we investigated whether ATF3 could be a target of STAT5 in the inhibition of adipogenic differentiation of BMSCs. To confirm the regulation of *Atf3* expression by STAT5 during adipogenic differentiation of BMSCs, the mRNA expression of *Atf3* was analyzed by real-time PCR during adipogenic differentiation under both STAT5 overexpression and STAT5-deficient conditions. We observed that the mRNA expression of *Atf3* was significantly upregulated and downregulated in STAT5-overexpressing and STAT5-deficient cells, respectively (Fig. 4a, b), which indicated that STAT5 regulates the mRNA expression of *Atf3* during adipogenic differentiation of BMSCs. To further examine whether STAT5 regulates the transcriptional activity of *Atf3*, a luciferase reporter assay and a ChIP assay were performed. The luciferase reporter assay revealed that the transcriptional activity of *Atf3* induced by thapsigargin was further induced by addition of STAT5 in a dose-dependent manner (Fig. 4c). Analysis of the *Atf3* promoter revealed several putative STAT5-binding sites located at positions −16, −239, and −852 in the *Atf3* promoter, but only the STAT5-binding site located at position −16 was valid for STAT5 binding (Supplementary Fig. 4a, b), and binding of STAT5 to the *Atf3* promoter was abolished in *Stat5* cKO BMSCs (Fig. 4d). Collectively, these results indicate that STAT5 upregulates the mRNA expression of *Atf3* by binding to the *Atf3* promoter to enhance its transcriptional activity.

If ATF3 is a target of STAT5 in the inhibition of adipogenic differentiation, it can be expected that ATF3 would exhibit effects similar to those of STAT5 on adipogenic differentiation. To test whether ATF3 overexpression and *Atf3* deficiency can mimic the inhibitory and promotive effects, respectively, of STAT5 overexpression and deficiency on adipogenic differentiation of BMSCs, adipogenic differentiation was examined under ATF3 overexpression and ATF3-deficient conditions. ATF3 overexpression inhibited adipogenic differentiation of BMSCs by suppressing the mRNA expression of *Pparɣ*, *Cebpɑ*, and *Fabp4* (Supplementary Fig. 5a–c). Next, *Atf3* was downregulated during adipogenic differentiation of BMSCs. Downregulation of *Atf3* using siRNA during adipogenic differentiation enhanced adipogenic differentiation by upregulating the mRNA expression of *Pparɣ*, *Cebpɑ*, and *Fabp4* (Supplementary Fig. 5d–f).

**Fig. 5:**
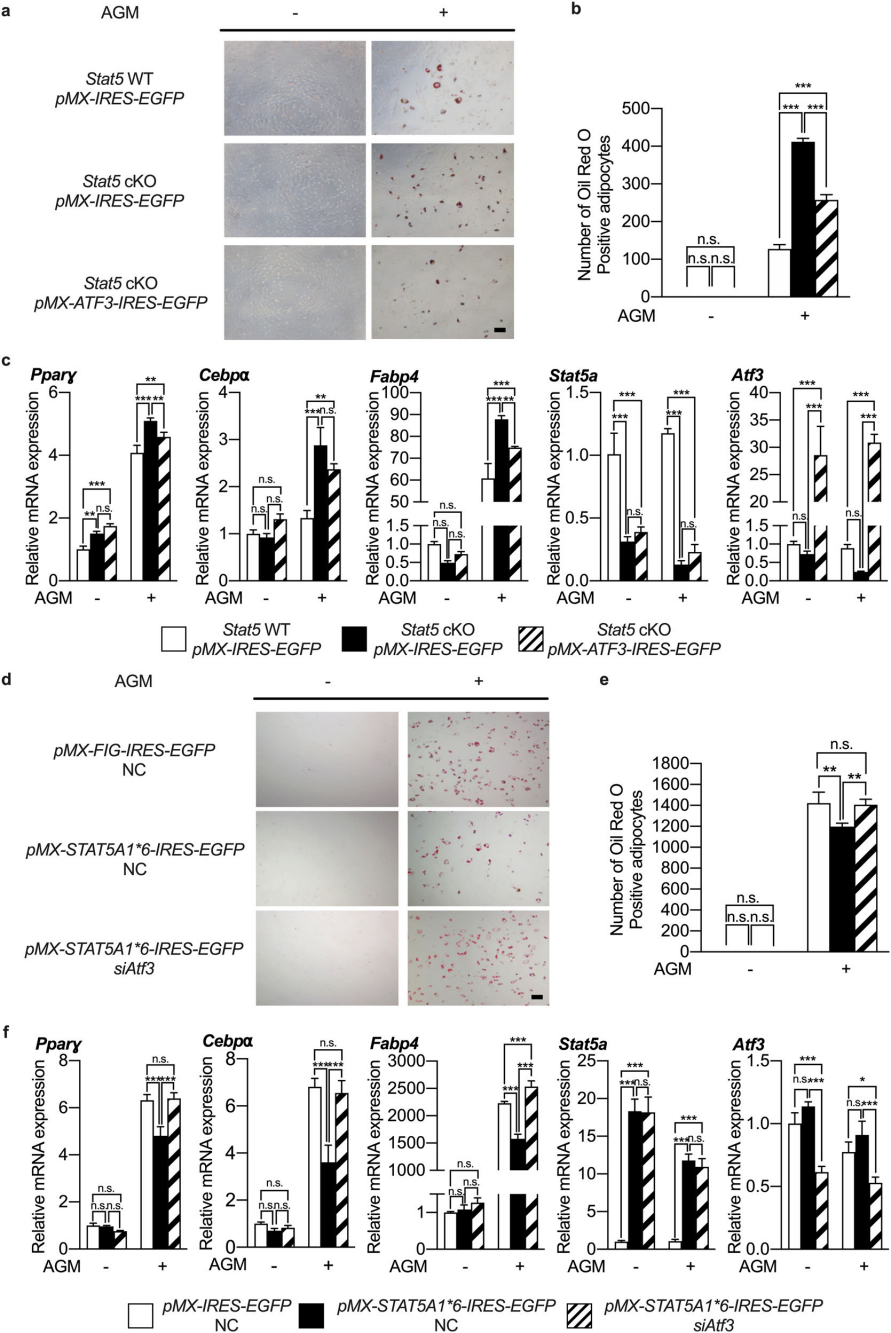
ATF3 mediates the inhibitory effect of STAT5 on adipocyte differentiation. **a–c** BMSCs were isolated from femurs and tibias of *Stat5* cKO mice and their wild-type littermates followed by transduction with control (*pMX-IRES-EGFP*) or ATF3 (*pMX-ATF3-IRES-EGFP*) retroviral vectors and were then cultured in the presence or absence of adipogenic differentiation factors (insulin, rosiglitazone, dexamethasone, and IBMX) for 5 days. **a** Cultured cells were stained using Oil Red O solution. **b** The number of Oil Red O-positive adipocytes was quantified. ***P < 0.001 vs. control; n.s. not significant. Statistical analyses were performed via ANOVA. **c** The mRNA levels of *Pparɣ*, *Cebpα*, *Fabp4*, *Stat5a*, and *Atf3* were assessed by quantitative real-time PCR. The data are presented as the mean ± SD of triplicate samples. **P < 0.01; ***P < 0.001 vs. control; n.s. not significant. Statistical analyses were performed via ANOVA. **d–f** BMSCs were isolated from femurs and tibias of wild-type mice followed by transfection with negative control siRNA (NC) or *Atf3* siRNA (si*Atf3*) after transduction with control (*pMX-IRES-EGFP*) or constitutively active STAT5A (pMX-STAT5A1*6-IRES-EGFP) retroviral vectors. Cells were cultured in the presence or absence of adipogenic differentiation factors (insulin, rosiglitazone, dexamethasone, and IBMX) for 5 days. **d** Cultured cells were stained using Oil Red O solution. **e** The number of Oil Red O-positive adipocytes was quantified. **P < 0.01 vs. control; n.s. not significant. Statistical analyses were performed via ANOVA. **f** The mRNA levels of *Pparɣ*, *Cebpα*, *Fabp4*, *Stat5a*, and *Atf3* were assessed by quantitative real-time PCR. The data are presented as the mean ± SD of triplicate samples. *P < 0.05; ***P < 0.001 vs. control; n.s. not significant. Statistical analyses were performed via ANOVA. AGM Adipogenic differentiation medium. Bars: (**a**) 200 µm; (**d**) 200 µm.

To investigate whether overexpression of ATF3 can restore the inhibitory effect of STAT5 on adipogenic differentiation of BMSCs, which was abrogated in *Stat5*-deficient cells, ATF3 was overexpressed during adipogenic differentiation of *Stat5* cKO adipocytes. The number of Oil Red O-positive adipocytes was significantly increased during adipogenic differentiation of *Stat5* cKO adipocytes, whereas ATF3 overexpression significantly reduced the number of *Stat5* cKO adipocytes, as evidenced by the Oil Red O staining results (Fig. 5a, b). This trend toward downregulation by ATF3 overexpression was also observed for the mRNA expression of adipogenic marker genes. The mRNA expression levels of *Pparɣ*, *Cebpɑ*, and *Fabp4* were significantly elevated in *Stat5* cKO adipocytes compared to wild-type adipocytes, while ATF3 overexpression in *Stat5* cKO adipocytes significantly abrogated the upregulation of adipogenic marker gene expression (Fig. 5c). To further determine the involvement of ATF3 in STAT5-mediated inhibition of adipogenic differentiation of BMSCs, *Atf3* was downregulated in STAT5-overexpressing adipocytes using *siAtf3*. STAT5 overexpression induced inhibition of adipogenic differentiation, as evidenced by the Oil Red O staining and real-time PCR results (Fig. 5d–f). However, the inhibition of adipogenic differentiation induced by STAT5 overexpression was abrogated when *Atf3* was simultaneously downregulated (Fig. 5f). These results collectively suggest that ATF3 is a target of STAT5 and mediates the inhibitory effect of STAT5 on adipogenic differentiation of BMSCs.

### Adipocyte-specific *Stat5* cKO mice exhibit reduced bone parameters

Having previously shown increased adiposity in the bone marrow of *Stat5* cKO mice in vivo (Fig. 3), we considered the possibility that the increased adiposity may affect the bone microenvironment, since adipocytes have the potential to affect the differentiation of osteoblasts and osteoclasts^34,35^.

**Fig. 6:**
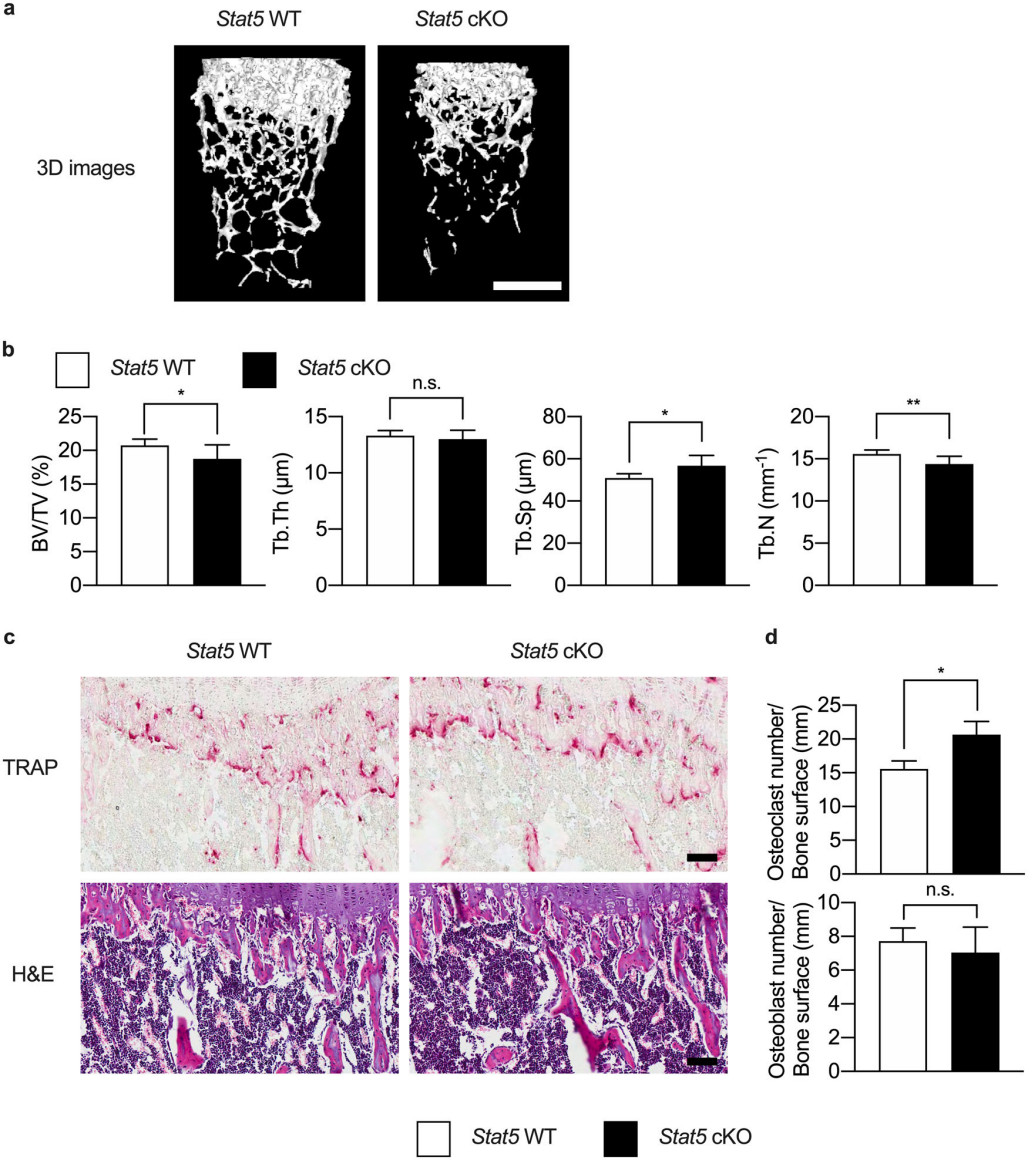
*Stat5* cKO mice exhibit reduced bone volume. **a**, **b** Femurs were isolated from *Stat5* cKO mice and their wild-type littermates and analyzed by µCT (*n* = 7 per group). **a** Representative 3D µCT images of femurs from *Stat5* cKO mice and their wild-type littermates. **b** Bone volume fraction, trabecular thickness, trabecular separation, and trabecular number were assessed from the µCT measurements. *P < 0.05; **P < 0.01 vs. control; n.s. not significant. Statistical analyses were performed via Student’s *t* test. **c**, **d** Tibias were harvested from *Stat5* cKO mice (*n* = 3) and their wild-type littermates (*n* = 4) and were subjected to histological analyses. **c** TRAP and H&E staining of histological sections of proximal tibias from *Stat5* cKO mice and their wild-type littermates. **d** Osteoclast number per bone surface and osteoblast number per bone surface were assessed based on TRAP and H&E staining, respectively. The data are presented as the mean ± SD values. *P < 0.05 vs. control; n.s. not significant. Statistical analyses were performed via Student’s *t* test. Bars: (**a**) 500 µm; (**c**) 100 µm.

Therefore, the bone phenotype was examined to determine whether the increased adiposity had any influence on the bone phenotype in adipocyte-specific *Stat5* cKO mice. Femurs and tibias were harvested from *Stat5* cKO mice and their wild-type littermates and analyzed by micro-CT and histological analyses. It was observed by micro-CT analysis that there were decreases in the bone volume fraction, trabecular thickness, and trabecular number, with concurrent increases in trabecular separation, in *Stat5* cKO mice compared to their wild-type littermates (Fig. 6a, b). Histological analysis was performed to further investigate the bone phenotype of *Stat5* cKO mice. *Stat5* cKO mice exhibited an increase in the osteoclast number, while there was no significant difference in the osteoblast number (Fig. 6c, d).

**Fig. 7:**
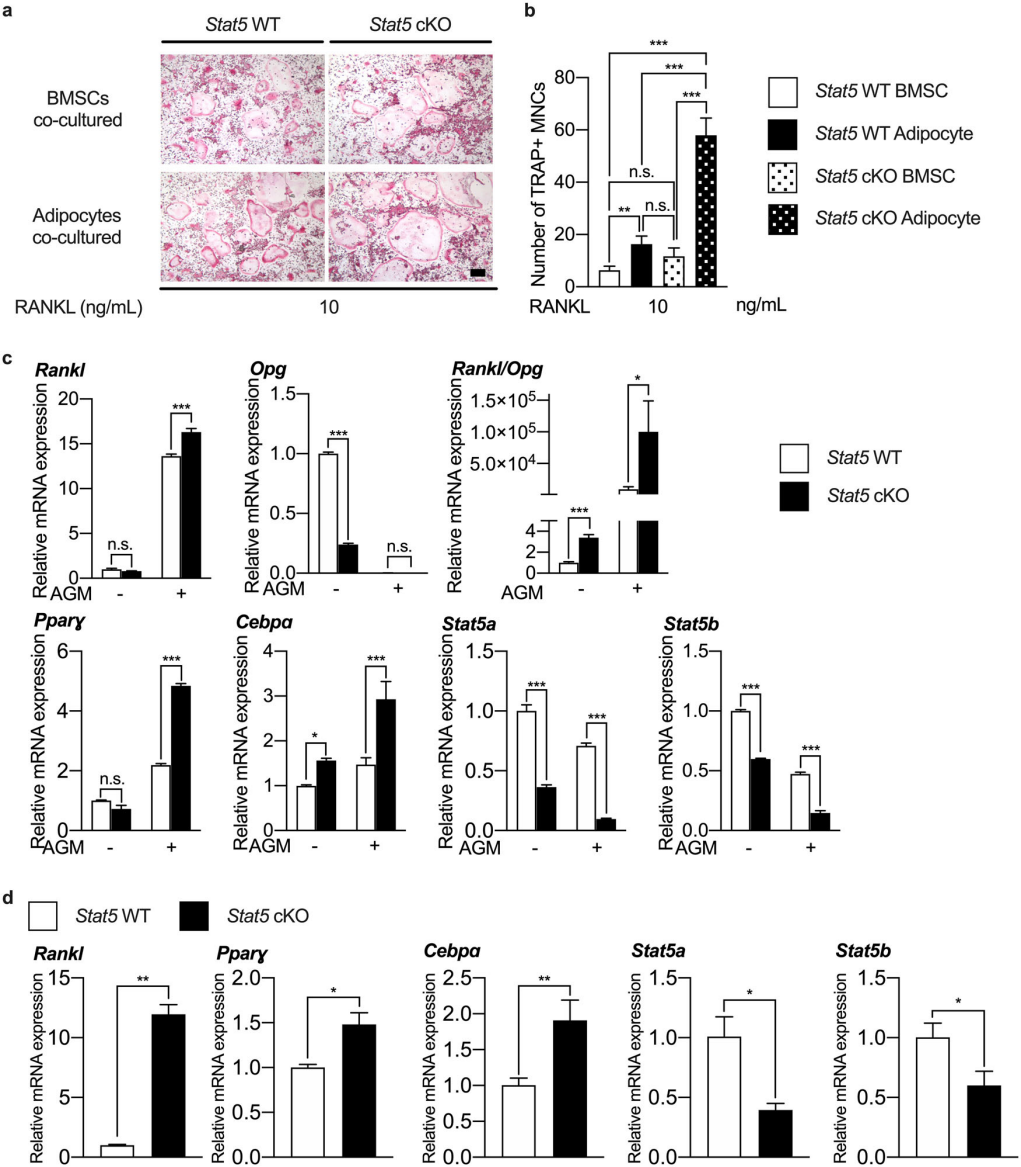
*Stat5*-deficient adipocytes promote RANKL-induced osteoclast differentiation of BMMs. **a**, **b** BMMs were isolated from 6-wk-old ICR mice and cocultured with *Stat5* WT BMSCs or adipocytes and *Stat5* cKO BMSCs or adipocytes in the presence of M-CSF and RANKL for 3 days. **a** TRAP staining of *Stat5* WT and cKO cells cocultured with osteoclasts. **b** Quantification of the number of TRAP-positive MNCs. **P < 0.01; ***P < 0.001 vs. control; n.s. not significant. Statistical analyses were performed via ANOVA. **c** BMSCs were isolated from 8-week-old *Stat5* cKO mice and their wild-type littermates and were differentiated into adipocytes in the presence of insulin, rosiglitazone, dexamethasone, and IBMX for 5 days. The mRNA expression levels of *Rankl*, *Opg*, *Rankl/Opg*, *Pparɣ*, *Cebpa*, *Stat5a*, and *Stat5b* were determined by quantitative real-time PCR. The data are presented as the mean ± SD of triplicate samples. *P < 0.05; ***P < 0.001 vs. control; n.s. not significant. Statistical analyses were performed via Student’s *t* test. **d** Bone marrow adipose cells were isolated from the long bones of 16-week-old *Stat5* cKO mice and their wild-type littermates. The mRNA expression levels of *Rankl*, *Pparɣ*, *Cebpɑ*, *Stat5a*, and *Stat5b* were determined by quantitative real-time PCR. The data are presented as the mean ± SD of triplicate samples. *P < 0.05; **P < 0.01 vs. control. Statistical analyses were performed via Student’s *t* test. AGM Adipogenic differentiation medium. Bar: (**a**) 100 µm.

*Stat5* cKO mice exhibited reduced bone parameters accompanied by an increase in the osteoclast number rather than a change in the osteoblast number; therefore, in vitro experiments were carried out to examine the possibility that *Stat5* cKO adipocytes may play a role in osteoclast differentiation. To this end, bone marrow-derived macrophages (BMMs) were cocultured with either *Stat5* cKO adipocytes or *Stat5* WT adipocytes and differentiated into osteoclasts. There was a significant increase in TRAP-positive multinuclear cells when BMMs were cocultured with *Stat5* cKO BMSCs, as evidenced by the TRAP staining results, and this increase in the TRAP-positive MNC number was even more significant when BMMs were cocultured with *Stat5* cKO adipocytes (Fig. 7a, b).

In addition, RANKL and OPG are the critical factors for osteoclast differentiation. To determine the factors facilitating osteoclast differentiation under coculture with *Stat5* cKO or *Stat5* WT adipocytes, the *Rankl* and *Opg* expression levels in *Stat5* cKO and *Stat5* WT adipocytes were determined by qPCR. qPCR revealed that the *Rankl*/*Opg* ratio was significantly increased in both BMSCs and adipocytes in the absence of *Stat5*. This increased ratio was due to increased *Rankl* and decreased *Opg* expression (Fig. 7c). Although the increased *Rankl* and decreased *Opg* expression observed in BMSCs and adipocytes was independent of genotype, the magnitude of the changes in the *Rankl* and *Opg* expression levels in each cell type was genotype-dependent. The *Rankl* expression level was comparable between *Stat5* cKO BMSCs and *Stat5* WT BMSCs but was significantly increased in adipocytes in the absence of *Stat5*. In addition, *Opg* expression was already downregulated in BMSCs with *Stat5* deficiency. Furthermore, *Opg* expression was dramatically decreased and almost disappeared after adipocyte differentiation. However, *Opg* expression was still lower in *Stat5* cKO adipocytes than in *Stat5* WT adipocytes (Fig. 7c). In addition to performing in vitro analyses of gene expression in adipocytes, we isolated bone marrow adipose cells from the long bones of *Stat5* cKO mice and their wild-type littermates to validate the contribution of *Stat5*-deficient adipocytes to the enhanced osteoclast formation in vivo. Bone marrow adipose cells isolated from the bone marrow of *Stat5* cKO mice exhibited downregulated *Stat5a* and *Stat5b* expression and upregulated *Pparɣ* and *Cebpɑ* expression, indicating the successful isolation of bone marrow adipose cells and substantiating the enhancement of adipocyte formation by *Stat5* deficiency in vivo (Fig. 7d). Furthermore, *Rankl* expression in *Stat5* cKO bone marrow adipose cells was substantially upregulated by *Stat5* deficiency, whereas *Opg* expression was undetectable in adipocytes of both genotypes in vivo. This finding further supports the contribution of *Stat5*-deficient adipocytes to increased osteoclastogenesis in vivo (Fig. 7d). Overall, these results suggest that knockdown of *Stat5* specifically in adipocytes enhances osteoclastogenic differentiation in vitro and in vivo by increasing the *Rankl*/*Opg* ratio; this increase is specifically due to increased *Rankl* and decreased *Opg* expression and is possibly substantial enough to affect the in vivo bone phenotype (Fig. 7c, d). Therefore, it can be deduced that adipocyte-specific *Stat5* cKO mice exhibit reduced bone mass due to the increased osteoclast number in addition to increased adiposity.

## Discussion

Although several investigations have been conducted, the role of STAT5 in adipocyte differentiation remains controversial and requires further clarification. In the present study, we revealed that STAT5 functions as a negative regulator of adipocyte differentiation, both in vitro and in vivo, using *Stat5*^*fl/fl*^*;Mx1-cre* and *Stat5*^*fl/fl*^*;Apn-cre* mice. Ectopic expression of constitutively active STAT5A inhibited adipocyte differentiation of BMSCs, while *Stat5* deficiency in BMSCs from *Stat5*^*fl/fl*^*;Mx1-cre* and *Stat5*^*fl/fl*^*;Apn-cre* mice was related to an increase in the bone marrow adipocyte number. In particular, adipocyte-specific *Stat5* deficiency resulted not only in increased adipocyte differentiation of BMSCs but also in enlargement and an increase in the mass of adipose tissues such as inguinal and retroperitoneal adipose tissues (Supplementary Fig. 3). Overall, these results suggest that STAT5 negatively regulates adipocyte differentiation of BMSCs and also adipocyte differentiation of MSCs.

In addition, there have been several clinical studies in human subjects that revealed the possibility that STAT5 negatively regulates adipocyte formation. Jorgensen et al.^36^ and Nielsen et al.^37^ reported phosphorylation of STAT5 by growth hormone (GH) in both adipose and muscle tissues in male human subjects. Furthermore, Bredella et al.^38^ observed amelioration of abdominal obesity accompanied by a diminished abdominal adipocyte size in human males upon GH treatment. These previous studies imply that phosphorylated STAT5 may negatively regulate adipocyte formation and contribute to obesity prevention, thereby strongly reinforcing the concept that STAT5 negatively regulates BMSC-derived adipocyte differentiation, as demonstrated in the present study.

The current study confirmed that STAT5 regulates adipocyte differentiation of BMSCs by regulating *Atf3* expression. To determine the target of STAT5 during adipocyte differentiation of BMSCs, the following criteria were considered: (1) gene expression contingent on the presence of STAT5 and (2) the ability to control adipocyte differentiation. With these criteria, *Atf3* was identified as a suitable gene. In our previous study, RNA sequencing revealed that *Atf3* expression was regulated by STAT5 in osteoclasts (GSE76988). Notably, ATF3 is known to be a negative regulator of adipocyte differentiation by inhibiting the expression of PPARɣ and C/EBPα and repressing PPARɣ-stimulated transactivation^32,33,39^. The present study demonstrated the regulation of *Atf3* expression by STAT5 during adipocyte differentiation of BMSCs under STAT5 overexpression and *Stat5*-deficient conditions and showed the inhibitory effect of ATF3 on adipocyte differentiation of BMSCs, consistent with our previous studies. In addition, ATF3 overexpression in *Stat5* cKO adipocytes ameliorated the aberrantly enhanced adipogenic differentiation of BMSCs, and the inhibitory effect of STAT5A1*6 on adipocyte differentiation was abolished upon siRNA*-*mediated *Atf3* silencing. Therefore, these results imply that ATF3 is a primary mediator of STAT5-dependent inhibition of BMSC differentiation into adipocytes and further reinforces the negative role of STAT5 in the differentiation of BMSC-derived adipocytes.

As shown in Fig. 5, ATF3 overexpression ameliorated the adipocyte differentiation of BMSCs promoted by *Stat5* deficiency. However, it did not completely reverse or abolish the enhanced adipocyte differentiation. Although STAT5 regulates adipogenic differentiation of BMSCs primarily via ATF3, the possibility that STAT5 may regulate adipocyte differentiation through other adipocyte regulatory factors cannot be excluded, and further studies are required to examine the mechanisms underlying these processes.

Regulation of adipocyte differentiation by STAT5 may influence bone homeostasis. Although the expression and function of STAT5 were preserved in *Stat5* cKO BMMs, *Stat5* cKO mice still exhibited decreased bone mass due to an increased osteoclast number. BMMs isolated from *Stat5* cKO mice exhibited RANKL-induced osteoclast differentiation comparable to that of BMMs isolated from *Stat5* WT mice, and differentiation of *Stat5* cKO osteoclasts revealed that the inhibitory effect of IL-3 on osteoclast differentiation was maintained, indicating that STAT5 functionality was preserved in *Stat5* cKO osteoclasts (Supplementary Fig. 6). In addition, *Rankl* expression was elevated in adipocytes, whereas *Opg* expression was downregulated or undetectable in adipocytes of both genotypes both in vitro and in vivo (Fig. 7c, d), in accordance with the findings of a previous study^40^. Takeshita et al.^40^ demonstrated that *Rankl* expression was upregulated but *Opg* expression was downregulated during adipocyte differentiation of preadipocytes. They also demonstrated that RANKL-expressing adipocytes were capable of generating osteoclasts from BMMs. Furthermore, Mori et al.^24^ reported that osteoclast differentiation is enhanced when precursor cells are cocultured with adipocytes, and this phenotype was reproduced in this study. Furthermore, when osteoclast precursor cells were cocultured with *Stat5* cKO adipocytes, there was a further increase in osteoclast differentiation relative to that observed when osteoclast precursor cells were cocultured with *Stat5* WT adipocytes. These results imply that the increased number of adipocytes in the bone marrow of *Stat5* cKO mice could mediate the reduction in bone volume by promoting osteoclast differentiation in vivo. Both the in vitro and in vivo results suggest that *Stat5* deficiency in adipocytes contributes not only to increased adipocyte differentiation but also to bone loss and suggest that the reduced bone volume in *Stat5*^*fl/fl*^*;Mx1-cre* mice observed in our previous study could be attributed to the increased number of adipocytes in the bone marrow of *Stat5*^*fl/fl*^*;Mx1-cre* mice.

The present study showed that STAT5 regulates the differentiation of BMSCs into adipocytes via ATF3. Furthermore, the results of our previous and present studies demonstrated that STAT5 regulates osteoclast differentiation directly through IL-3 and indirectly through the regulation of adipocyte differentiation to participate in the maintenance of bone homeostasis. Therefore, regulation of *Stat5* could constitute a new therapeutic approach, especially for postmenopausal osteoporosis and obesity-induced osteoporosis, since STAT5 plays a critical role in both adipocytes and osteoclasts.

## Supplementary information

Supplementary Information

## References

[1] Cui Y (2004). Inactivation of Stat5 in mouse mammary epithelium during pregnancy reveals distinct functions in cell proliferation, survival, and differentiation. Mol. Cell Biol..

[2] Gouilleux F, Wakao H, Mundt M, Groner B (1994). Prolactin induces phosphorylation of Tyr694 of Stat5 (MGF), a prerequisite for DNA binding and induction of transcription. EMBO J..

[3] Lin JX (2012). Critical Role of STAT5 transcription factor tetramerization for cytokine responses and normal immune function. Immunity.

[4] Nosaka T (1999). STAT5 as a molecular regulator of proliferation, differentiation and apoptosis in hematopoietic cells. EMBO J..

[5] Groner B (2000). Regulation of the trans-activation potential of STAT5 through its DNA-binding activity and interactions with heterologous transcription factors. Growth Horm. IGF Res..

[6] Kazansky AV, Kabotyanski EB, Wyszomierski SL, Mancini MA, Rosen JM (1999). Differential effects of prolactin and src/abl kinases on the nuclear translocation of STAT5B and STAT5A. J. Biol. Chem..

[7] Darnell JE (1997). STATs and gene regulation. Science.

[8] Decker T, Kovarik P, Meinke A (1997). GAS elements: a few nucleotides with a major impact on cytokine-induced gene expression. J. Interferon Cytokine Res.

[9] Teglund S (1998). Stat5a and Stat5b proteins have essential and nonessential, or redundant, roles in cytokine responses. Cell.

[10] Lee J (2016). STAT5 is a key transcription factor for IL-3-mediated inhibition of RANKL-induced osteoclastogenesis. Sci. Rep..

[11] Jung HS (2012). Peroxisome proliferator-activated receptor γ/signal transducers and activators of transcription 5A pathway plays a key factor in adipogenesis of human bone marrow-derived stromal cells and 3T3-L1 preadipocytes. Stem Cells Dev..

[12] Kawai M (2007). Growth hormone stimulates adipogenesis of 3T3-L1 cells through activation of the Stat5A/5B-PPARγ pathway. J. Mol. Endocrinol..

[13] Nanbu-Wakao R (2002). Stimulation of 3T3-L1 adipogenesis by signal transducer and activator of transcription 5. Mol. Endocrinol..

[14] Wakao H, Wakao R, Oda A, Fujita H (2011). Constitutively active Stat5A and Stat5B promote adipogenesis. Environ. Health Prev. Med.

[15] Baik M (2017). Muscle-specific deletion of signal transducer and activator of transcription 5 augments lipid accumulation in skeletal muscle and liver of mice in response to high-fat diet. Eur. J. Nutr..

[16] Baik M (2016). Liver-specific deletion of the signal transducer and activator of transcription 5 gene aggravates fatty liver in response to a high-fat diet in mice. J. Nutr. Biochem..

[17] Kaltenecker D (2017). Adipocyte STAT5 deficiency promotes adiposity and impairs lipid mobilisation in mice. Diabetologia.

[18] Zhao L, Wang A, Corl BA, Jiang H (2014). Effect of growth hormone on the differentiation of bovine preadipocytes into adipocytes and the role of the signal transducer and activator of transcription 5b. J. Anim. Sci..

[19] Tanaka Y, Nakayamada S, Okada Y (2005). Osteoblasts and osteoclasts in bone remodeling and inflammation. Curr. Drug Targets Inflamm. Allergy.

[20] Simonet WS (1997). Osteoprotegerin: a novel secreted protein involved in the regulation of bone density. Cell.

[21] Kong YY (1999). OPGL is a key regulator of osteoclastogenesis, lymphocyte development and lymph-node organogenesis. Nature.

[22] Justesen J (2001). Adipocyte tissue volume in bone marrow is increased with aging and in patients with osteoporosis. Biogerontology.

[23] Rosen CJ, Bouxsein ML (2006). Mechanisms of disease: is osteoporosis the obesity of bone?. Nat. Clin. Pract. Rheumatol..

[24] Mori K (2014). Potentiation of osteoclastogenesis by adipogenic conversion of bone marrow-derived mesenchymal stem cells. Biomed. Res.

[25] Kang S (2007). Wnt signaling stimulates osteoblastogenesis of mesenchymal precursors by suppressing CCAAT/enhancer-binding protein alpha and peroxisome proliferator-activated receptor γ. J. Biol. Chem..

[26] Moerman EJ, Teng K, Lipschitz DA, Lecka-Czernik B (2004). Aging activates adipogenic and suppresses osteogenic programs in mesenchymal marrow stroma/stem cells: the role of PPAR-γ2 transcription factor and TGF-beta/BMP signaling pathways. Aging Cell.

[27] Liu LF, Shen WJ, Zhang ZH, Wang LJ, Kraemer FB (2010). Adipocytes decrease Runx2 expression in osteoblastic cells: roles of PPARγ and adiponectin. J. Cell Physiol..

[28] Tropel P (2004). Isolation and characterisation of mesenchymal stem cells from adult mouse bone marrow. Exp. Cell Res..

[29] Eguchi J (2011). Transcriptional control of adipose lipid handling by IRF4. Cell Metab..

[30] Onishi M (1998). Identification and characterization of a constitutively active STAT5 mutant that promotes cell proliferation. Mol. Cell. Biol..

[31] Yang J (2020). The increase in bone resorption in early-stage type I diabetic mice is induced by RANKL secreted by increased bone marrow adipocytes. Biochem. Biophys. Res. Commun..

[32] Jang MK, Jung MH (2014). ATF3 represses PPARγ expression and inhibits adipocyte differentiation. Biochem. Biophys. Res. Commun..

[33] Jang MK, Kim CH, Seong JK, Jung MH (2012). ATF3 inhibits adipocyte differentiation of 3T3-L1 cells. Biochem. Biophys. Res. Commun..

[34] Goto H (2011). Human bone marrow adipocytes support dexamethasone-induced osteoclast differentiation and function through RANKL expression. Biomed. Res..

[35] Hozumi A (2009). Bone marrow adipocytes support dexamethasone-induced osteoclast differentiation. Biochem. Biophys. Res. Commun..

[36] Jorgensen JO (2006). GH receptor signaling in skeletal muscle and adipose tissue in human subjects following exposure to an intravenous GH bolus. Am. J. Physiol. Endocrinol. Metab..

[37] Nielsen C (2008). Growth hormone signaling in vivo in human muscle and adipose tissue: impact of insulin, substrate background, and growth hormone receptor blockade. J. Clin. Endocrinol. Metab..

[38] Bredella MA (2017). GH administration decreases subcutaneous abdominal adipocyte size in men with abdominal obesity. Growth Horm. IGF Res..

[39] Jang MK, Jung MH (2015). ATF3 inhibits PPARγ-stimulated transactivation in adipocyte cells. Biochem. Biophys. Res. Commun..

[40] Takeshita S, Fumoto T, Naoe Y, Ikeda K (2014). Age-related marrow adipogenesis is linked to increased expression of RANKL. J. Biol. Chem..

