# Supplementary material for: Alternative regulatory mechanism for the maintenance of bone homeostasis via STAT5-mediated regulation of the differentiation of BMSCs into adipocytes
Source: Exp Mol Med. 2021 May 14;53(5):848–63. doi: 10.1038/s12276-021-00616-9 (PMC8178345; doi:10.1038/s12276-021-00616-9)
Supplement: Supplementary file 1 — Supplementary Information [file 12276_2021_616_MOESM1_ESM.docx]

**SUPPLEMENTARY INFORMATION**

**
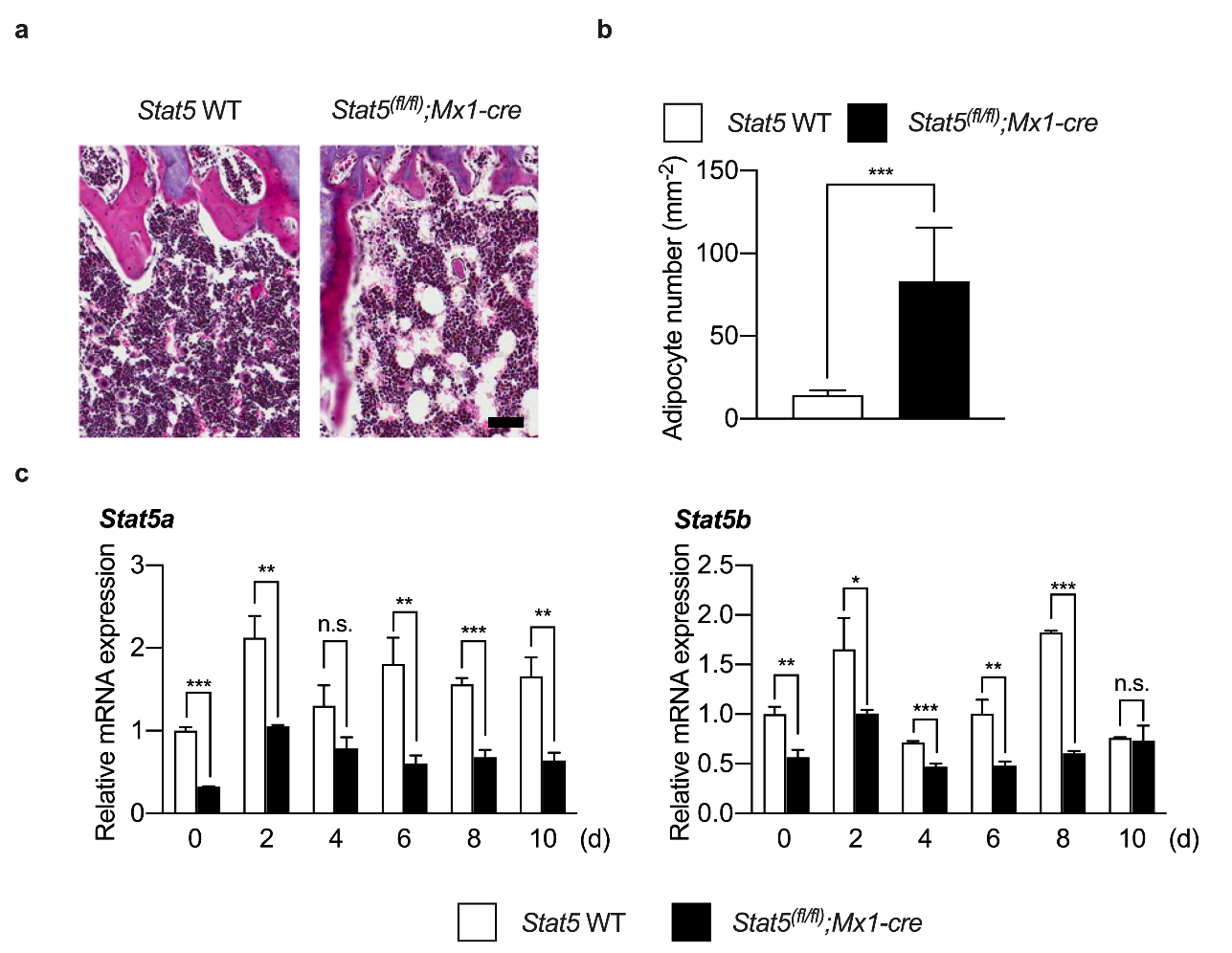
**

**Supplementary Figure 1. Adipocyte formation is increased in *Stat5^fl/fl^;Mx1-cre* mice.** **(a-b)** Tibiae were isolated from *Stat5^fl/fl^;Mx1-cre* mice (n = 7) or wild-type littermates (n = 5) and subjected to histological analysis. **(a)** Representative images of hematoxylin & eosin stained tibiae isolated from *Stat5^fl/fl^;Mx1-cre* mice or wild-type littermates. **(b)** Quantification of adipocyte number in the bone marrow of *Stat5^fl/fl^;Mx1-cre* mice or wild-type littermates based on hematoxylin & eosin staining. ***P < 0.001 vs. control. Statistical analyses were performed via t-test. **(c)** BMSCs were isolated from *Stat5^fl/fl^;Mx1-cre* mice or wild-type littermates and cultured in the presence or absence of adipogenic differentiation factors (insulin, rosiglitazone, dexamethasone, and IBMX) for the indicated days. Subsequently, mRNA levels of *Stat5a* and *Stat5b* were assessed by quantitative real-time PCR. Data represent the mean ± SD of triplicate samples. *P < 0.05; **P < 0.01; ***P < 0.001 vs. control; n.s. not significant. Statistical analyses were performed via t-test. Bar: **(a)** 100 µm.


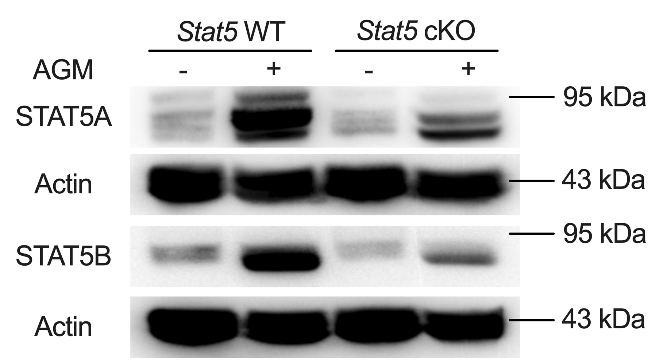


**Supplementary Figure 2. Knockdown efficiency of *Stat5^fl/fl^;Apn-cre* on the STAT5A and STAT5B levels during the adipogenic differentiation of BMSCs.** Bone marrow mesenchymal stem cells (BMSCs) were isolated from *Stat5^fl/fl^;Apn-cre* mice or wild-type littermates and cultured in the presence or absence of adipogenic differentiation factors (insulin, rosiglitazone, dexamethasone, and IBMX) for 8 days. Subsequently, whole cell lysates were harvested from cultured cells and were immunoblotted with the indicated antibodies. All gels were run under the same experimental conditions; the representative images are cropped and shown. AGM: adipogenic differentiation medium.


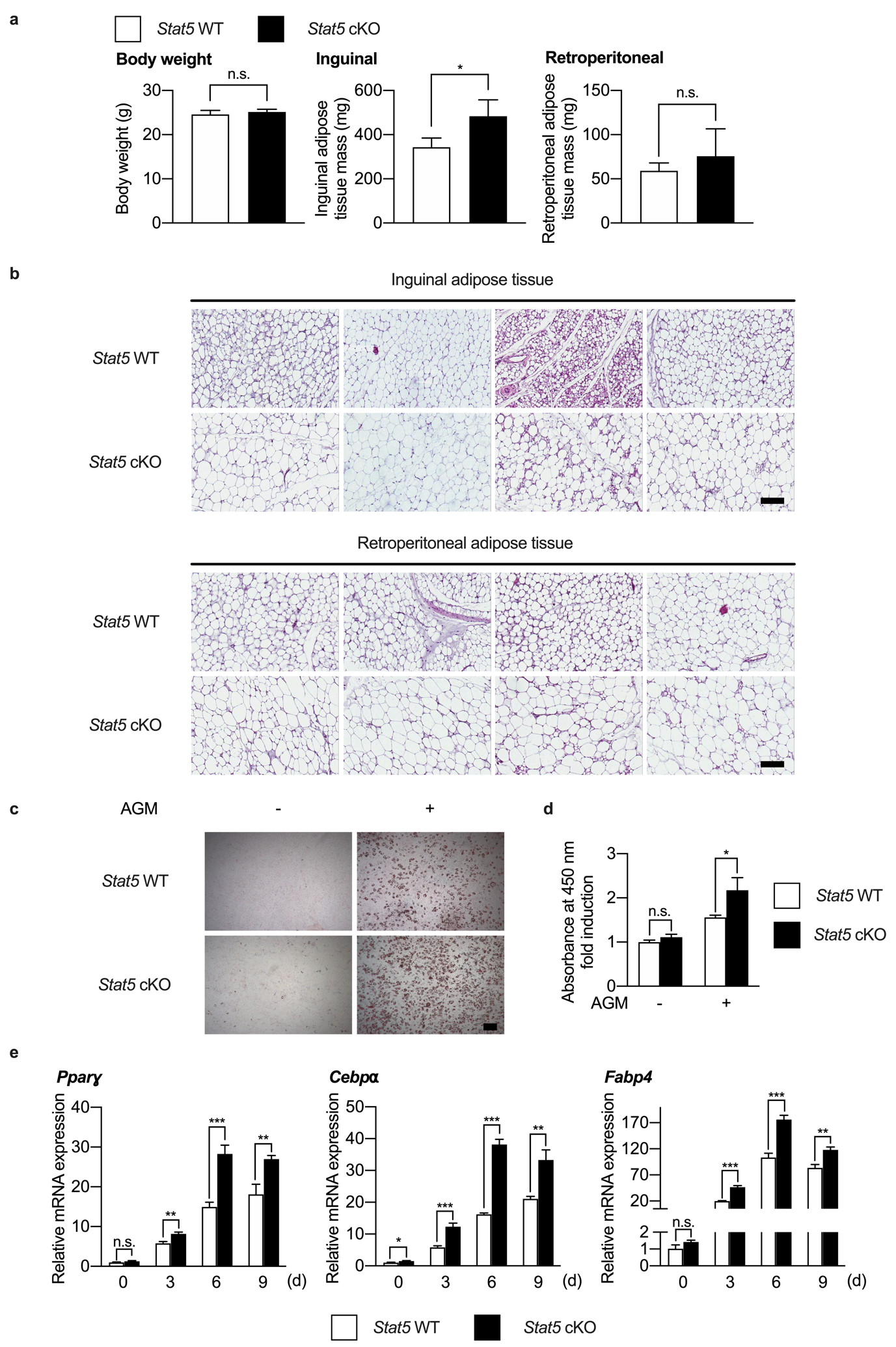


**Supplementary Figure 3. Adiposity is increased in adipose tissues from *Stat5* cKO mice.** **(a)** Measurement of body weight, inguinal adipose tissue, and retroperitoneal adipose tissue of *Stat5* cKO mice or wild-type littermates (n = 4 per group). *P < 0.05 vs. control; n.s. not significant. Statistical analyses were performed via t-test. **(b)** Representative images of hematoxylin & eosin-stained inguinal adipose tissues and retroperitoneal adipose tissues isolated from *Stat5* cKO mice or wild-type littermates (n = 4 per group). **(c-e)** Adipose tissue-derived mesenchymal stem cells (ADSCs) were isolated from inguinal adipose tissues from *Stat5* cKO or wild-type littermates and cultured in the presence or absence of adipogenic differentiation factors (insulin, rosiglitazone, dexamethasone, and IBMX) for the indicated days. **(c)** Cultured cells were stained using Oil Red O staining solution. **(d)** Oil Red O-positive adipocytes were quantified by isopropanol extraction. *P < 0.05 vs. control; n.s. not significant. Statistical analyses were performed via t-test. **(e)** mRNA levels of *Pparɣ*, *Cebp⍺*, and *Fabp4* were assessed by quantitative real-time PCR. Data represent the mean ± SD of triplicate samples. *P < 0.05; **P < 0.01; ***P < 0.001 vs. control; n.s. not significant. Statistical analyses were performed via t-test. AGM: Adipogenic differentiation medium. Bars: **(b)** 100 µm; **(c)** 200 µm.

**
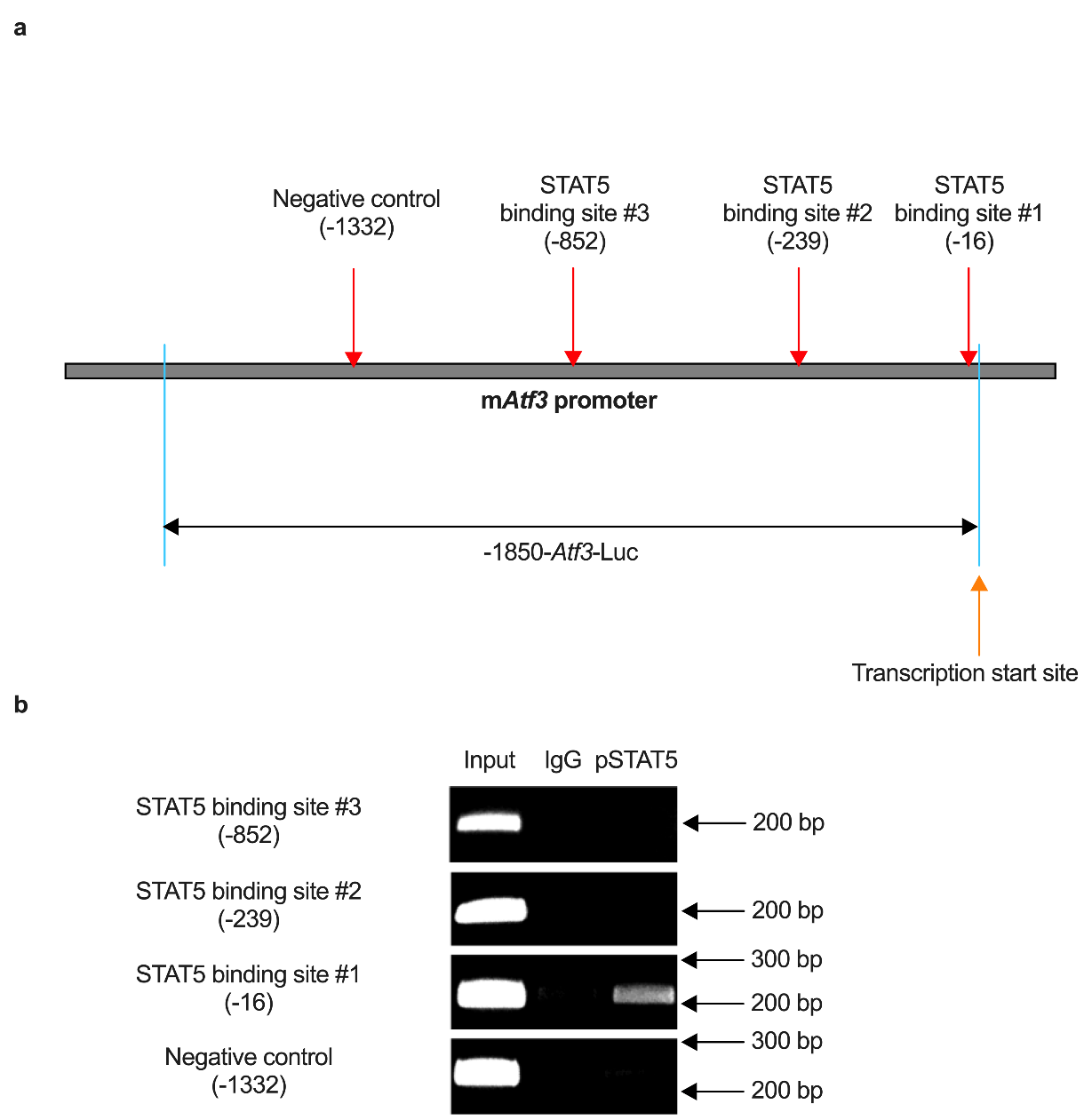
**

**Supplementary Figure 4. The *Atf3* promoter contains STAT5-binding sites. (a)** Illustration of m*Atf3* promoter with putative STAT5-binding sites indicated. **(b)** BMSCs were isolated from *Stat5* WT mice and immunoprecipitated with pSTAT5 antibody or IgG as a negative control. Precipitated DNA was subjected to PCR with 3 different primers targeting 3 different putative STAT5-binding sites (-16, -239, and -852) and negative control primer targeting -1332.


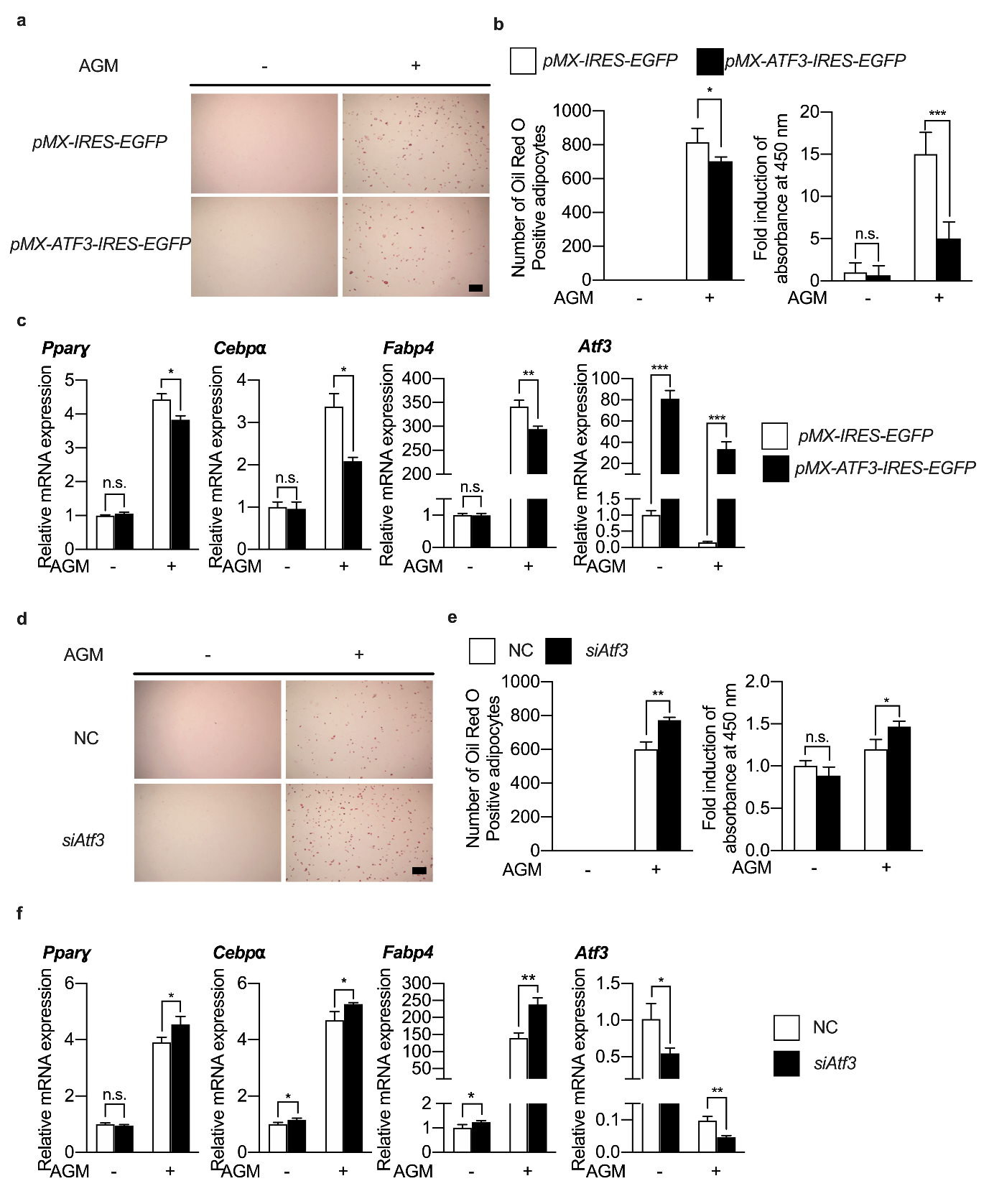


**Supplementary Figure 5. ATF3 functions as a negative regulator of adipocyte differentiation.** **(a-c)** BMSCs were transduced with control (*pMX-IRES-EGFP*) or ATF3 (*pMX-ATF3*) retrovirus and cultured in the presence or absence of adipogenic differentiation factors (insulin, rosiglitazone, dexamethasone, and IBMX) for 5 days. **(a)** Cultured cells were stained using Oil Red O staining solution. **(b)** Number of Oil Red O-positive cells were counted and quantified by isopropanol extraction. *P < 0.05; ***P < 0.001 vs. control; n.s. not significant. Statistical analyses were performed via t-test. **(c)** mRNA levels of *Pparɣ*, *Cebp⍺*, and *Fabp4* were assessed by quantitative real-time PCR. Data represent the mean ± SD of triplicate samples. *P < 0.05; **P < 0.01; ***P < 0.001 vs. control; n.s. not significant. Statistical analyses were performed via t-test. **(d-f)** BMSCs were transfected with negative control siRNA (NC) or *Atf3* siRNA (*siAtf3*) and cultured in the presence or absence of adipogenic differentiation factors (insulin, rosiglitazone, dexamethasone, and IBMX) for 5 days. **(d)** Cultured cells were stained using Oil Red O staining solution. **(e)** Number of Oil Red O-positive cells were counted and quantified by isopropanol extraction. *P < 0.05; **P < 0.01 vs. control; n.s. not significant. Statistical analyses were performed via t-test.­_­_­­­­­ **(f)** mRNA levels of *Pparɣ*, *Cebp⍺*, and *Fabp4* were assessed by quantitative real-time PCR. Data represent the mean ± SD of triplicate samples. *P < 0.05; **P < 0.01 vs. control; n.s. not significant. Statistical analyses were performed via t-test. AGM: Adipogenic differentiation medium. Bars: **(a)** 200 µm; **(d)** 200 µm.


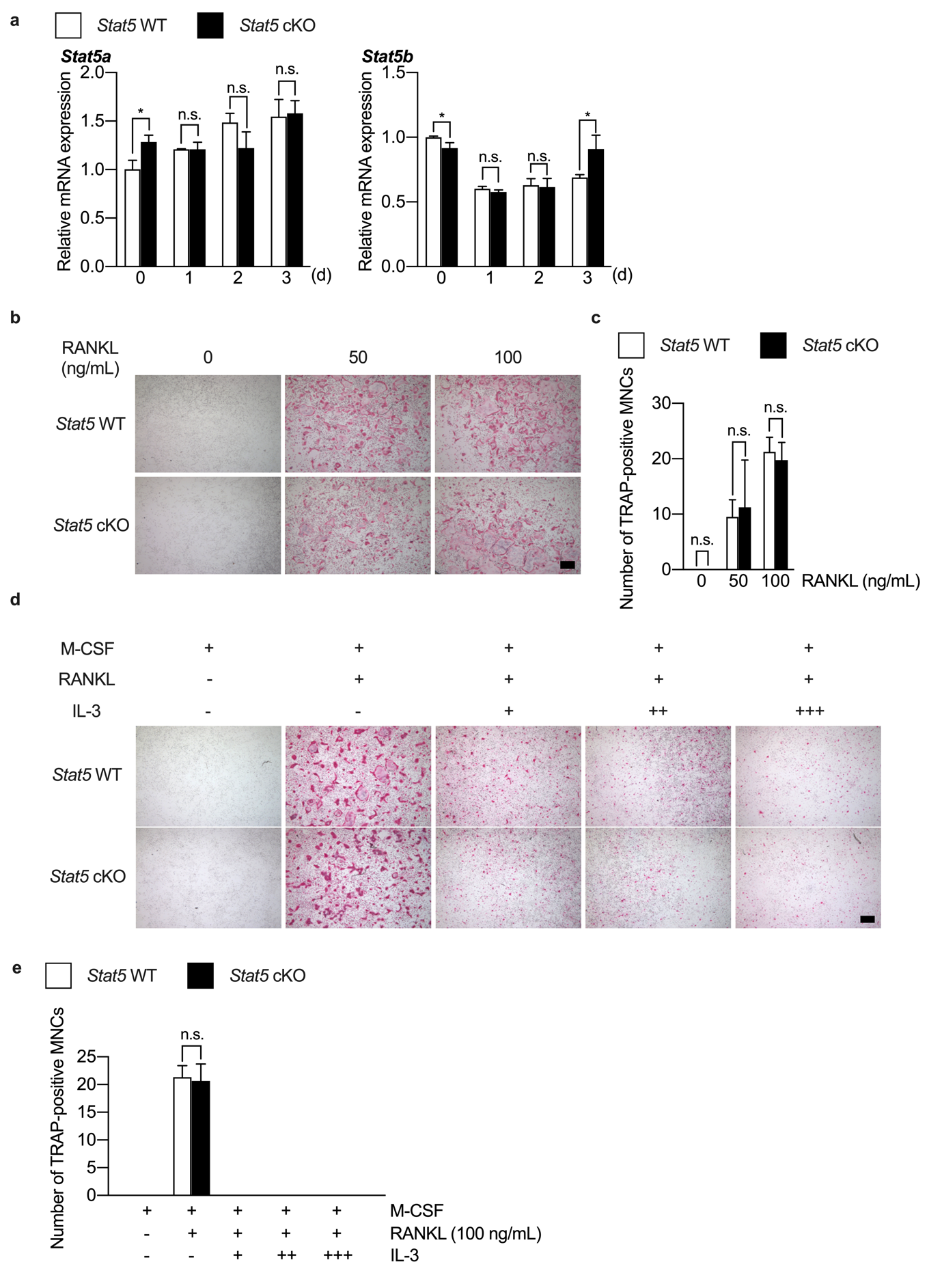


**Supplementary Figure 6. Function of *Stat5* is preserved in *Stat5^fl/fl^;Apn-cre* BMMs.** **(a-c)** BMMs were isolated from *Stat5* cKO mice or wild-type littermates and cultured for three days in the presence of M-CSF and RANKL. **(a)** mRNA levels of *Stat5a* and *Stat5b* were assessed by quantitative real-time PCR. Data represent the mean ± SD of triplicate samples. *P < 0.05 vs. control; n.s. not significant. Statistical analyses were performed via t-test. **(b)** TRAP staining of *Stat5* WT or cKO osteoclasts. **(c)** Quantification of number of TRAP-positive MNCs. n.s. not significant. Statistical analyses were performed via t-test. **(d-e)** BMMs were isolated from *Stat5* cKO mice or wild-type littermates and cultured for three days in the presence of M-CSF and RANKL with or without IL-3. **(d)** TRAP staining of *Stat5* WT or cKO osteoclasts. **(e)** Quantitation of the number of TRAP-positive MNCs. n.s. not significant. Statistical analyses were performed via t-test. Bars: **(b)** 200 µm; **(d)** 200 µm.
